# Identification and epidemiological evaluation of gastric cancer risk factors: based on a field synopsis and meta-analysis in Chinese population

**DOI:** 10.18632/aging.203484

**Published:** 2021-09-06

**Authors:** Fujiao Duan, Chunhua Song, Jiachen Shi, Peng Wang, Hua Ye, Liping Dai, Jianying Zhang, Kaijuan Wang

**Affiliations:** 1 College of Public Health, Zhengzhou University, Zhengzhou, Henan Province, China; 2 Key Laboratory of Tumor Epidemiology of Henan Province, Zhengzhou, Henan Province, China; 3 Department of Internal Medicine, The First Affiliated Hospital of Zhengzhou University,Zhengzhou, Henan Province, China; 4 Medical Research Office, Affiliated Cancer Hospital of Zhengzhou University, Zhengzhou, Henan Province, China

**Keywords:** gastric cancer, risk factors, genetic variants, susceptibility, field synopsis

## Abstract

To summarize and assess the credibility and strength of non-genetic factors and genetic variation on gastric cancer risk, we performed a field synopsis and meta-analysis to identify the risk of gastric cancer in Chinese population. Cumulative evidence was graded according to the Venice criteria, and attributable risk percentage (*ARP*) and population attributable risk percentage (*PARP*) were used to evaluate the epidemiological effect. A total of 956 studies included non-genetic (404 studies) and genetic factors (552 studies) were quantified, and data on 1161 single nucleotide polymorphisms (SNPs) were available. We identified 14 non-genetic factors were significantly associated with gastric cancer risk. For the analysis of time trends, *H. pylori* infection rate in gastric cancer and population showed a downward trend. Meanwhile 22 variants were identified significantly associated with gastric cancer: 3 (*PLCE1* rs2274223, *PSCA* rs2976392, *MUC1* rs4072037) were high and 19 SNPs were intermediate level of summary evidence, respectively. For non-genetic factors, the top three for *ARP* were 54.75% (pickled food), 65.87% (stomach disease), and 49.75% (smoked and frying). For *PARP* were 34.22% (pickled food), 34.24% (edible hot food) and 23.66%(*H. pylori* infection). On the basis of *ARP* and *PARP* associated with SNPs of gastric cancer, the top three for *ARP* were 53.91% (*NAT2*, rs1799929),53.05% (*NAT2* phenotype), and 42.85% (*IL-10*, rs1800896). For *PARP* (Chinese Han in Beijing) were 36.96% (*VDR*, rs731236), 25.58% (*TGFBR2*, rs3773651) and 20.56% (*MUC1*, rs4072037). Our study identified non-genetic risk factors and high-quality biomarkers of gastric cancer susceptibility and their contribution to gastric cancer.

## INTRODUCTION

Cancer is a major public health problem in the world [1]. Gastric cancer is a highly lethal malignancy worldwide, being the fourth most common cancer and the second leading cause of cancer-related mortality [2]. Among the total cancer mortality rates, male and female gastric cancer mortality rates accounted for 30% and 20%, respectively [3]. It is concerning that Eastern Asia had the highest estimated morbidity and mortality rates worldwide [4]. In China, gastric cancer is the second most commonly diagnosed cancer among men, second only to lung cancer [5]. Although surgical techniques, radiotherapy and chemotherapy regimens have helped reduce the incidence and mortality rates of gastric cancer [6] the overall 5-year survival rate is still only approximately 25% [7, 8].

The pathogenesis of gastric cancer represents a typical example of gene-environment interaction [9]. Among environmental factors, unhealthy eating habits or behavior and Helicobacter pylori (*H. pylori*) infection are the most common causes of gastric cancer. Because of the functional variation of genes, genetic factors play a crucial role in the development of gastric cancer, leading to cancer outcomes [10].

In the past two decades, several strategies have been used to determine the genetic determinants of gastric cancer, including *H. pylori* eradication, precancerous lesions, genome wide association study (GWAS) and identification of candidate genes. Moreover, based on GWAS results, about 11 sites in the human genome have been reported to link with the development of gastric cancer in Chinese population [11].

In actuality, before the emergence and widespread use of genome-wide scanning, hundreds of case-control studies have detected candidate polymorphisms (mainly based on rational selection of Biology) in gastric cancer. Although some of these associations showed hope, almost all of them failed to replicate [12]. In order to explore the role of genetic variation in the occurrence of gastric cancer, a large number of studies have been carried out, and genetic variation are considered as potential risk factors of gastric cancer. Interestingly, different DNA variants appear to be associated with the risk of specific sites of gastric cancer. However, the results of these studies are not always consistent. So far, no systematic review covering all polymorphisms and non-genetic factors has been published in Chinese population, The aim of this study is to fill this gap in medical research literature on Chinese population from international and Chinese public databases by presenting the first systematic review of the available evidence in field of non-genetic (*H. pylori* infection, family history, behavioral factors (ie, smoking, drinking and diet, et al.), mental depression and other factors (ie, obesity, hypertension and diabetes)) and genetic factors (single nucleotide polymorphisms, SNPs) and the risk of gastric cancer, including the evaluation of their epidemiological significance.

## RESULTS

### Study identification

**Figure 1:**
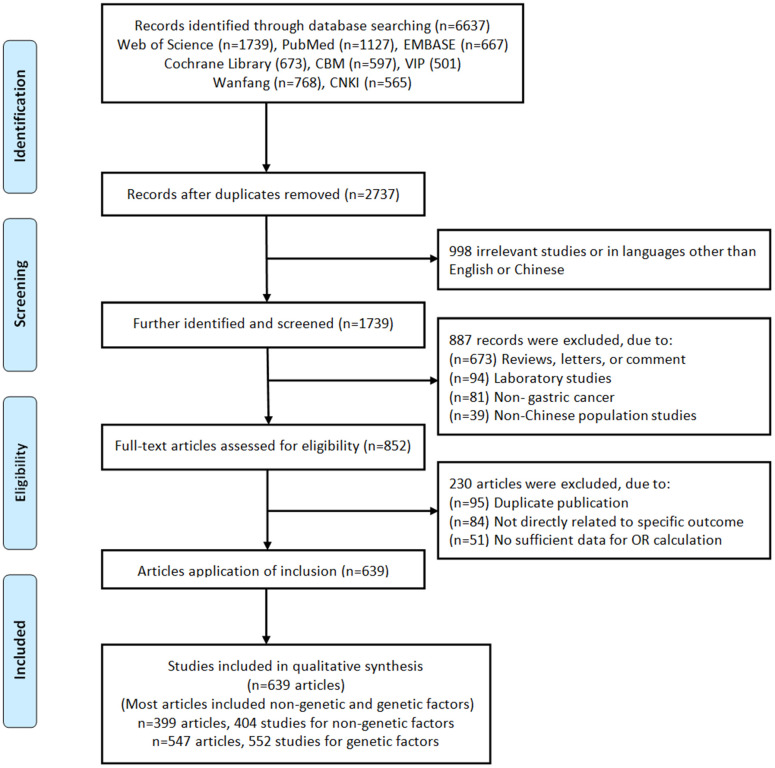
Flow chart of literature search and study selection.

A flow diagram of the literature search strategy was summarized in Figure 1. Based on the search strategy, a total of 6,637 records were retrieved. Of these, 3,900 articles were excluded as duplicates, 998 articles were excluded as irrelevant (not related to risk factors or genetic variants). After screening the titles and abstracts, the remaining 852 eligible articles were assessed for full-text review. Due to most independent studies included non-genetic and genetic factors associated with gastric cancer, and therefore 639 articles (n=399 articles, 404 studies for non-genetic factors; n=547 articles, 552 studies for genetic factors) eligible articles were included in qualitative synthesis (Supplementary References). If an article contains two populations, we consider it as two independent studies. The SNP was eliminated if the number of studies involving it was less than 3. As a result, a total of 70 SNPs involving 48 genes were available. We calculated the Hardy-Weinberg equilibrium (HWE) based on the control genotyping data provided by the eligible study, and 114 studies (14 SNPs involved 10 genes) were excluded due to the non-HWE. Therefore, 56 SNPs involved 38 genes were quantitative synthesis.

### Baseline characteristics of eligible studies

Non-genetic factors data set from 404 studies containing 161,960 cases and 218,270 controls and genetic factors data set from 552 studies providing genotype data on 844,632 cases and 1,101,997 controls of Chinese population Based on the design, most of the data sets of non-genetic factors (263/399, 65.91%) and genetic factors (65.04%, 359/552) are derived from population-based case-control articles. 378 articles (69.10%, 378/547) was randomly repeated a portion of samples as quality control while genotyping to evaluate reproducibility and accuracy.

A total of 16 non-genetic factors were extracted, namely *H. pylori* infection, smoking, drinking, family history, stomach disease, high salt diet, pickled food, fast eating, irregular meals, edible hot food, smoked and frying, spicy diet, mental depression, BMI, Hypertension, Diabetes.

For genetic factors, partial inclusion studies specified the histological subtypes (intestinal, diffuse or mix) (10.07%, 59/552) of gastric cancer, the site (cardia and non-cardia) (15.04%, 83/552) of the primary gastric cancer and *H. pylori* infection status (positive vs negative) (13.22%, 73/552).

**Figure 2:**
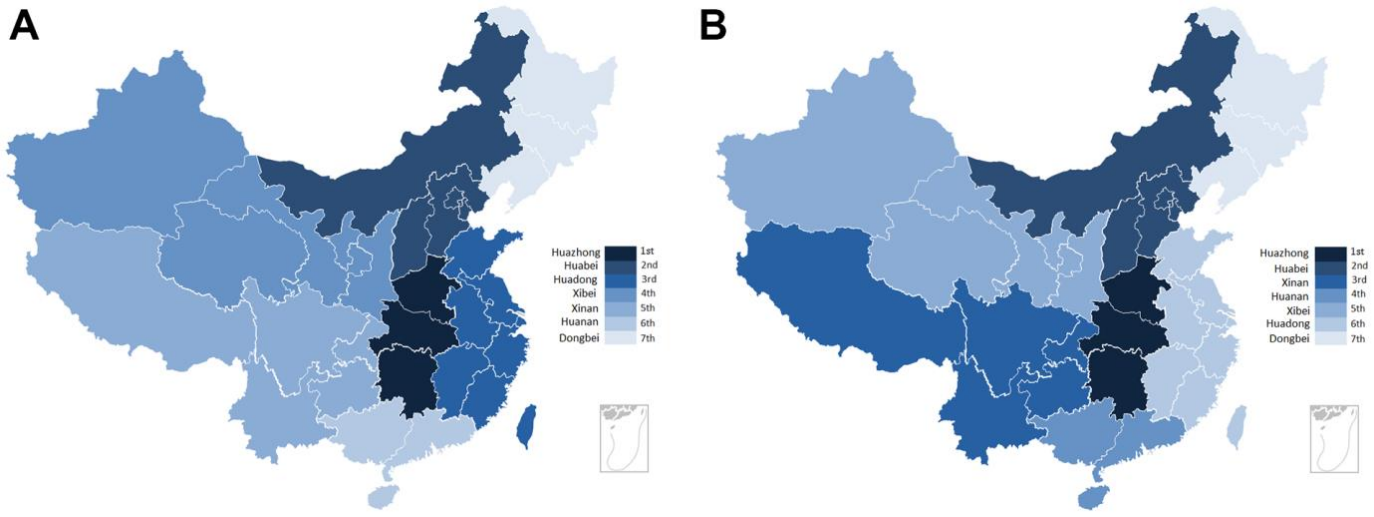
**The distribution of *H. pylori* infection rate for gastric cancer and population in each province.** (**A**) The regional distribution of *H. pylori* infection for population in China. (**B**) The regional distribution of *H. pylori* infection for gastric cancer in China.

PubMed was used to identify GWAS associated with gastric cancer, resulting in a total of three GWASs (11 SNPs) [13–15], the11 SNPs were all located in 5 genes. Overall, data on 1161 polymorphisms involving 460 distinct genes were available. Most SNPs were located on chromosomes 1 and 8. The number of articles from Jiangsu Province was the highest (218), followed by Shandong (38) and Henan (37) Province. The distribution of *H. pylori* infection in the general population and gastric cancer based on China's regional division was shown in Figure 2A, 2B.

### Quantitative synthesis findings

**Figure 3:**
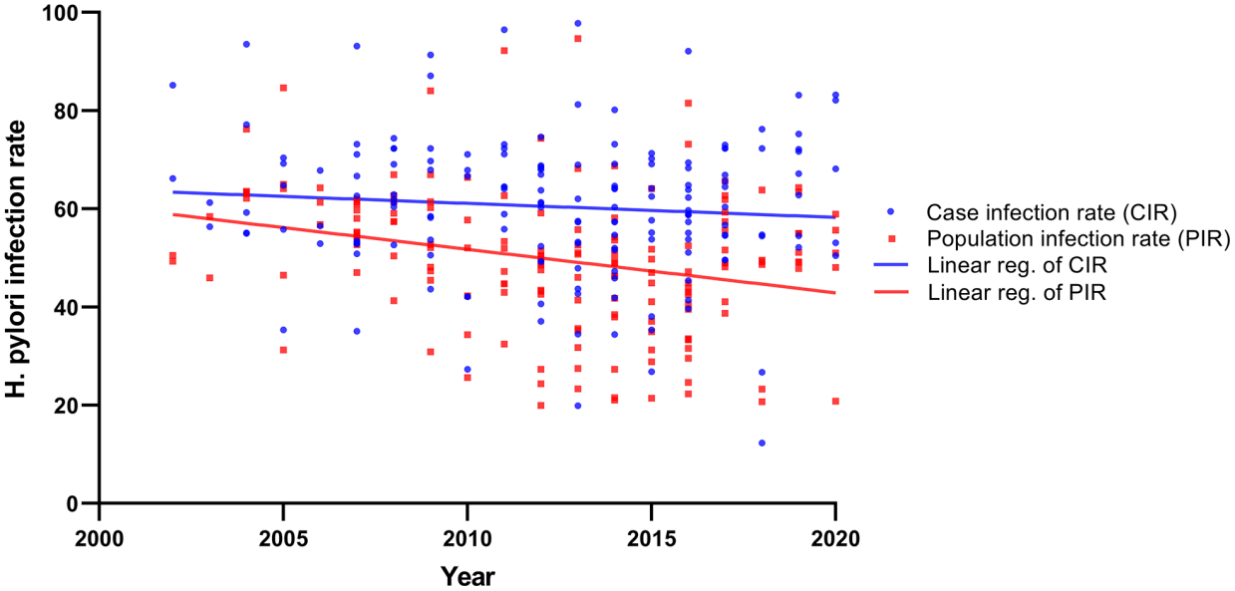
The linear regression analysis showed that from 2000 to 2020, H. pylori infection rate in gastric cancer and population.

Demographic and environmental risk factors for gastric cancer were summarized in Table 1. *H. pylori* infection, smoking, drinking, family history, stomach disease, high salt diet, pickled food, fast eating, irregular meals, edible hot food, smoked and frying, spicy diet and mental depression were significantly associated with gastric cancer risk. Of note, the results showed that the risk of gastric cancer in diabetic patients was lower than that in the general population (*OR* = 0.76, *95% CI*: 0.68-0.84), while hypertension (*OR* = 0.95, *95% CI*: 0.88-1.01) and obesity (BMI, *OR* = 0.79, *95% CI*: 0.53-1.21) were not associated with gastric cancer. According to the type of gastric cancer, the results showed that the risk factors for cardia and non-cardia gastric cancers were different (Table 1). Meanwhile, linear regression analysis showed that from 2000 to 2020, *H. pylori* infection rate showed a slowly downward trend in gastric cancer and population, and it was relatively obvious in the population (Figure 3).

**Table 1:** Main combined results of non-genetic factors based on fixed or random.

**Factors**	**Case** **n/N**	**Control** **n/N**	***OR* (*95CI%*)**	***P***	***Z***	**Model**	**Studies**
***Gastric cancer***							
*Hp*-infection	29728/49542	30896/62375	1.62(1.47,1.79)	<0.001	9.81	Random	153
Smoking	49978/111107	59897/142863	1.28(1.22,1.34)	<0.001	9.01	Random	286
Drinking	36481/100199	42928/129380	1.29(1.23,1.37)	<0.001	8.79	Random	253
Family history	3068/40960	7759/52405	1.87(1.81,1.93)	<0.001	31.54	Random	112
Stomach disease	3834/12784	2717/19727	2.93(2.32,2.71)	<0.001	9.28	Random	38
High salt diet	3600//7138	4651/11921	1.74(1.64,1.86)	<0.001	15.17	Random	31
Pickled food	2952/5416	3540/8164	2.21(2.03,2.38)	<0.001	18.17	Random	28
Fast eating	2650/5350	2583/7402	1.83(1.71,2.01)	<0.001	14.13	Random	18
Irregular meals	3040/8449	3910/12107	1.71(1.62,1.91)	<0.001	12.86	Random	28
Edible hot food	2479/5593	3349/8720	2.37(1.66,2.81)	<0.001	5.73	Random	20
Smoked and frying	744/3708	1191/5677	1.99(1.46,2.70)	<0.001	4.41	Random	15
Spicy diet	1177/3612	1022/4522	1.73(1.16,2.63)	0.008	2.97	Random	12
Mental depression	1990/5909	1631/7333	1.82(1.34,2.46)	<0.001	3.87	Random	19
BMI	2866/10654	5323/13475	0.79(0.53,1.21)	0.21	1.22	Random	23
Hypertension	2020/8493	2236/8963	0.95(0.88,1.01)	0.10	1.52	Fixed	24
Diabetes	845/8283	1082/8364	0.76(0.68,0.84)	<0.001	5.14	Fixed	23
***Non-cardiac Cancer***							
*Hp*-infection	559/1035	925/1979	1.46(1.24,1.72)	<0.001	4.59	Fixed	6
Smoking	1376/2693	2335/4662	1.24(1.12,1.37)	<0.001	4.15	Fixed	13
Drinking	2384/6799	3231/10271	1.39(1.09,1.77)	0.008	2.65	Random	12
Family history	418/2060	612/3918	3.28(1.75,6.13)	<0.001	3.72	Random	10
Irregular meals	191/417	97/490	3.15(1.59,6.24)	0.001	3.30	Random	3
***Cardiac Cancer***							
*Hp*-infection	897/1345	1178/2282	1.80(1.55,2.09)	<0.001	0.14	Fixed	5
Smoking	3741/7476	5583/12650	1.36(1.15,1.61)	<0.001	3.53	Random	24
Drinking	1697/5445	2277/9529	1.45(1.29,1.63)	<0.001	6.37	Random	18
Family history	529/1864	596/2852	2.46(1.25,4.85)	0.009	2. 61	Random	5

Based on the number of studies, the number of studies ranged from 3 to 23, the top five genetic variations were the following: *GSTM1* (1p13.3), *GSTT1* (22q11.23), *MTHFR* (rs1801133), *XRCC1* (rs1799782) and *XRCC1* (rs25487).

**Figure 4:**
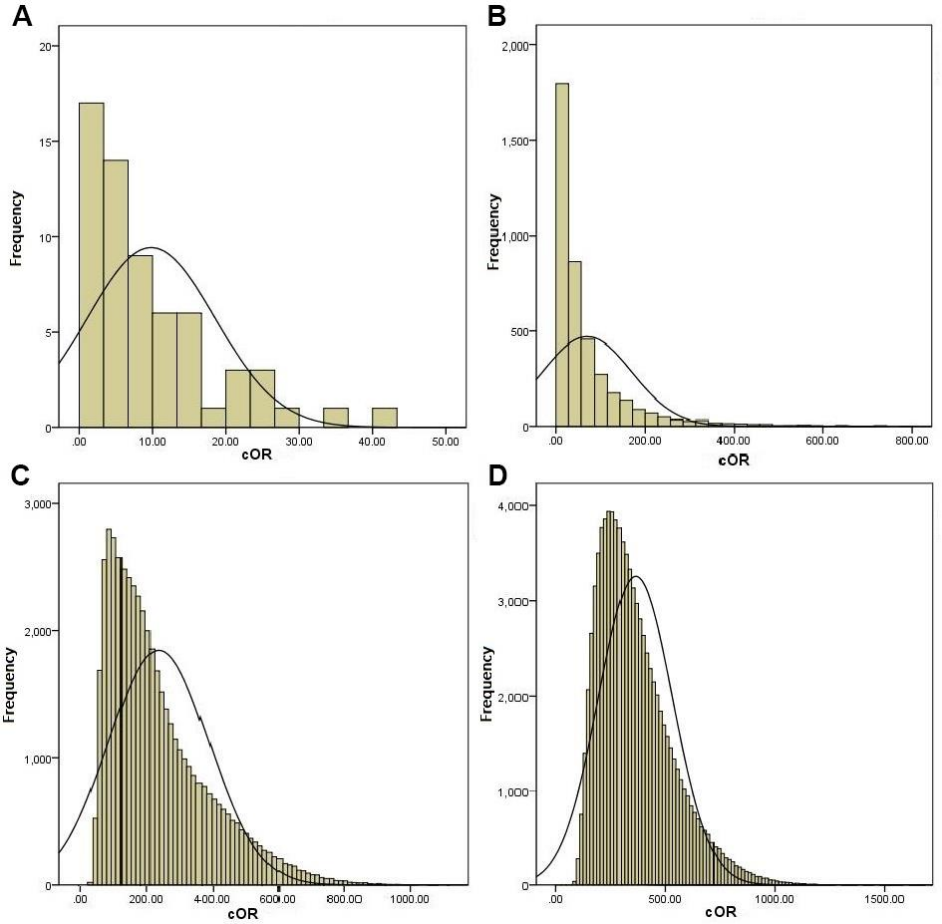
**The distribution of genetic variants that were significantly related to the risk of gastric cancer based on the main function of genes.** (**A**) Inflammatory related genes (*COX-2* rs20417(C), *IL-17A* rs2275913, *IL-17A* rs8193036, *TGFBR2* rs3773651, *IL-10* rs1800896 and *VDR* rs731236). (**B**) B+Metabolic pathway related genes (*MTHFR* rs1801133, *NAT2* rs1799930, *NAT2* rs1799929, *NAT2* phenotype, *GSTM1* and *GSTT1*). (**C**) A+B+Signal pathway related gene (*PLCE1* rs2274223, *PLCE1* rs3765524, *PSCA* rs2294008, *PSCA* rs2976392, *PRKAA1* rs13361707 and *MUC1* rs4072037). (**D**) A+B+C+Base excision repair pathway related gene (*XRCC1* rs1799782, *XRCC1* rs25487, *XRCC3* rs861539 and *ERCC5* rs751402).

Among the significant associations identified by this synopsis, the level of summary evidence 3(13.64%) (*PLCE1* rs2274223, *PSCA* rs2976392, *MUC1*rs4072037) were high and 19 (83.36%) (*MTHFR* rs1801133, *COX-2* rs20417, *XRCC1* rs1799782 and rs25487, *XRCC3* rs861539, *NAT2* rs1799930 and rs1799929, *PLCE1* rs3765524, GSTM1, GSTT1IL-17A rs2275913 and rs8193036, *PSCA* rs2294008, *PRKAA1* rs13361707, *ERCC5* rs751402, *TGFBR* rs3773651, *IL-10* rs1800896 and *VDR* rs731236) were intermediate level of summary evidence, respectively. The default value of FPRP critical value was 0.5, and the prior probability was set as 0.1, 0.01 and 0.001 respectively. Among the high-quality correlations, FPRP was the best for 22/22, 19/22 and 6/22 at 0.1, 0.01 and 0.001 levels, respectively. The details of significant associations characterized by a high or intermediate level of pooled evidence were summarized in Table 2. According to the main functions of genes, the distribution of genetic variants significantly related to the risk of gastric cancer were shown in Figure 4A–4D. As we add more susceptible genes into the model, the risk distribution gradually expands, and the population can better distinguish between high-risk and low-risk categories.

**Table 2:** Meta-analysis results: genetic variants significantly associated with gastric cancer risk with a high or intermediate level of summary evidence.

**Gene**	**Variant ID**	**Chr**	**Risk** **allele**	**RAF of** **control**	**Participants**	***OR*(*95%CI*)**	***P*-Value**	**Venice criteria^a^**	**Evidence level^b^**	**FPRP**	**FPRP**	**FPRP**
										**(0.1)**	**(0.01)**	**(0.001)**
*COX-2*	rs20417	1	C	0.044	1237	1.48(1.04,2.11)	0.03	ABB	Intermediate	0.356	0.859	0.954
*IL-17A*	rs2275913	6	A	0.386	9335	1.28(1.14,1.44)	<0.001	ABA	Intermediate	0.001	0.007	0.065
*IL-17A*	rs8193036	4	T/CT	0.420	6120	1.13(1.01,1.26)	0.03	ABA	Intermediate	0.200	0.734	0.965
*TGFBR2*	rs3773651	3	G	0.311	2923	1.40(1.22,1.61)	<0.001	ABA	Intermediate	0.051	0.371	0.856
*IL-10*	rs1800896	1	G/TT	0.056	2534	4.40(1.36,12.62)	0.02	BBB	Intermediate	0.321	0.586	0.940
*VDR*	rs731236	12	T	0.936	4202	1.61(1.32,1.96)	<0.001	AAB	Intermediate	0.000	0.001	0.011
*MTHFR*	rs1801133	1	T	0.410	7432	1.19(1.31,1.27)	<0.001	ABB	Intermediate	0.000	0.000	0.000
*NAT2*	rs1799930	8	A	0.171	3274	1.43(1.27,1.62)	<0.001	AAB	Intermediate	0.000	0.000	0.000
*NAT2*	rs1799929	8	T	0.119	3274	2.17(1.58,2.98)	<0.001	ABB	Intermediate	0.000	0.000	0.000
*NAT2*		8	Slow	0.084	3275	2.13(1.71,2.64)	<0.001	ABA	Intermediate	0.000	0.000	0.000
*GSTM1*		1	Null	0.488	9971	1.32(1.21,1.43)	<0.001	BBB	Intermediate	0.000	0.000	0.000
*GSTT1*		2	Null	0.476	7747	1.25(1.07,1.45)	0.004	ABA	Intermediate	0.025	0.243	0.764
*PLCE1*	rs2274223	10	A	0.226	9710	1.30(1.23,1.37)	<0.001	AAA	High	0.000	0.000	0.000
*PLCE1*	rs3765524	10	T	0.206	1950	1.31(1.19,1.44)	<0.001	BBB	Intermediate	0.006	0.067	0.418
*PRKAA1*	rs13361707	5	C	0.477	13743	1.42(1.35,1.48)	<0.001	ABA	Intermediate	0.001	0.008	0.077
*PSCA*	rs2294008	8	T	0.276	10202	1.19(1.12,1.27)	<0.001	ABA	Intermediate	0.000	0.000	0.000
*PSCA*	rs2976392	8	A	0.243	7316	1.13(1.05,1.22)	<0.001	AAA	High	0.000	0.002	0.023
*MUC1*	rs4072037	1	A	0.824	5521	1.31(1.21,1.43)	<0.001	AAA	High	0.000	0.000	0.001
*XRCC1*	rs1799782	19	T	0.276	7770	1.40(1.22,1.61)	<0.001	BBA	Intermediate	0.000	0.000	0.002
*XRCC1*	rs25487	19	A	0.292	6887	1.11(1.04,1.20)	0.004	ABA	Intermediate	0.001	0.009	0.082
*XRCC3*	rs861539	14	T	0.141	3054	1.29(1.12,1.50)	<0.001	BAB	Intermediate	0.097	0.243	0.779
*ERCC5*	rs751402	13	A	0.325	3899	1.21(1.10,1.33)	<0.001	BAB	Intermediate	0.003	0.035	0.269

^a^Venice criteria: A (high), B (moderate), C (weak) credibility for three parameters (amount of evidence, heterogeneity and bias; see text for more details); ^b^level of evidence: overall level of summary evidence according to the Venice criteria. Chr, chromosome; RAF, Risk Allele Frequency of the control; FPRP, false positive report probability (three levels of prior probability, see text for more details); HP, *Helicobacter pylori*; LL, 95% lower level; sOR, summary OR; UL, 95% upper level.

Association between the genetic variants and risk of gastric cancer in different comparisons were shown in Table 3.

**Table 3:** Association between the genetic variants and risk of gastric cancer in different comparisons.

**Gene rs#**	**Per-allele**	**Heterozygous**	**Homozygous**	**Dominant model**	**Recessive model**
	***OR*(*95%CI*)**	***OR*(*95%CI*)**	***OR*(*95%CI*)**	***OR*(*95%CI*)**	***OR*(*95%CI*)**
*COX-2* rs20417	1.48(1.04, 2.11)	1.48(1.03, 2.15)	3.74(0.15,92.29)	1.51(1.05, 2.19)	0.69(0.48,1.01)
*IL-17A* rs2275913	1.28 (1.14, 1.44)	1.23(1.12, 1.35)	1.65 (1.29, 2.11)	1.30(1.19, 1.42)	1.45 (1.16, 1.82)
*IL-17A* 8193036	1.03(0.95,1.12)	1.13(1.01, 1.25)	0.92(0.76,1.12)	1.09(0.99,1.21)	0.87(0.72,1.06)
*TGFBR2* rs3773651	1.40(1.22, 1.61)	1.42(0.89,2.26)	1.99 (1.26, 3.12)	1.80(1.15, 2.82)	1.45(1.23,1.69)
*IL-10* rs1800896	1.75(0.89,3.41)	2.04(0.86,4.20)	4.40(1.36, 12.62)	1.39(0.95,1.83)	3.99(1.23, 11.28)
*VDR* rs731236	1.61 [1.32, 1.96]	0.60 [0.49, 0.74]	1.23(0.41,3.66)	0.60 [0.49, 0.74]	1.17(0.39,3.48)
*MTHFR* rs1801133	1.19(1.13, 1.27)	1.32(1.19, 1.45)	1.36(1.19, 1.53)	1.33(1.21, 1.46)	1.18(1.07, 1.32)
*NAT2* rs1799929	2.17 (1.58, 2.98)	2.18(1.43, 3.31)	6.92(3.79, 12.63)	2.38 (1.57, 3.61)	5.76 (3.14, 10.55)
*NAT2* rs1799930	1.43(1.27, 1.62)	1.15(0.99,1.35)	2.85 (2.05, 3.97)	1.33 (1.15, 1.53)	2.72(1.96, 3.78)
*NAT2* phenotype^a^	2.13 (1.71, 2.64)	-	-	-	-
*GSTM1* 1p13.3^b^	1.32(1.21, 1.43)	-	-	-	-
*GSTT1* 22q11.23^c^	1.25 (1.04, 1.45)	-	-	-	-
*PLCE1* rs2274223	1.30(1.23, 1.37)	1.22(1.05, 1.42)	1.64 (1.37, 1.91)	1.70 (1.00, 2.86)	1.40 (1.03, 1.90)
*PLCE1* rs3765524	1.31(1.19, 1.44)	1.37(1.13, 1.65)	1.37(0.88,21.4)	1.37(1.14, 1.64)	1.22(0.79,1.90)
*PRKAA1* rs13361707	1.42 (1.35, 1.48)	1.50 (1.37, 1.63)	2.03 (1.84, 2.23)	1.66 (1.53, 1.80)	1.53 (1.42, 1.66)
*PSCA* rs2294008	1.19(1.12, 1.27)	1.30 (1.20, 1.42)	1.19(0.86,1.64)	1.30 (1.13, 1.49)	0.92(0.81,1.06)
*PSCA* rs2976392	1.13(1.05, 1.22)	1.24(1.13, 1.37)	1.12(0.94,1.34)	1.22 (1.12, 1.34)	1.02(0.86,1.20)
*MUC1* rs4072037	1.31(1.21, 1.43)	0.98(0.72,1.33)	1.35(1.00, 1.82]	1.24(0.92,1.66)	1.31(1.06, 1.61]
*XRCC1* rs1799782	1.40(1.22, 1.61)	1.44(1.23, 1.68)	1.64(1.39, 1.93)	1.51(1.28, 1.78)	1.45(1.01, 2.07)
*XRCC1* rs25487	1.11(1.04, 1.20)	1.22(1.10, 1.34)	1.11(0.93,1.32)	1.20(1.09, 1.32)	1.00(0.85,1.19)
*XRCC3* rs861539	1.29(1.12, 1.50)	1.30(1.09, 1.56)	1.74(1.15, 2.63)	1.33(1.12, 1.58)	1.33(0.89,1.99)
*ERCC5* rs751402	1.21(1.10, 1.33)	1.20 (1.05, 1.38)	1.48 (1.20, 1.82)	1.25 (1.10, 1.43)	1.34(1.11, 1.64)

^a^The *NAT2* rapid and slow acetylator phenotypes; ^b,c^Null versus Normal.

### Sensitivity analysis and publication bias

We conducted the sensitivity analysis by omitting each study in turn on the pooled ORs, and removing any of the included studies, there was no significant impact on the pooled outcomes, which indicated the pooled OR was stable (Data not shown). The publication bias of the included studies was evaluated through Begger’s and Egger’s tests. The results indicated no evidence of publication bias.

Begger’s (*t*=0.69, *P*=0.466 for non-genetic factors; *t*=1.44, *P*=0.163 for genetic factors) and Egger’s test (*t*=0.93, *P*=0.367, 95CI: -0.359, 1.203 for non-genetic factors; *t*=0.69, *P*=0.131, 95CI: -0.122, 1.1317 for genetic factors) were used to evaluate the publication bias.

### Epidemiological evaluation of risk factors

**Figure 5:**
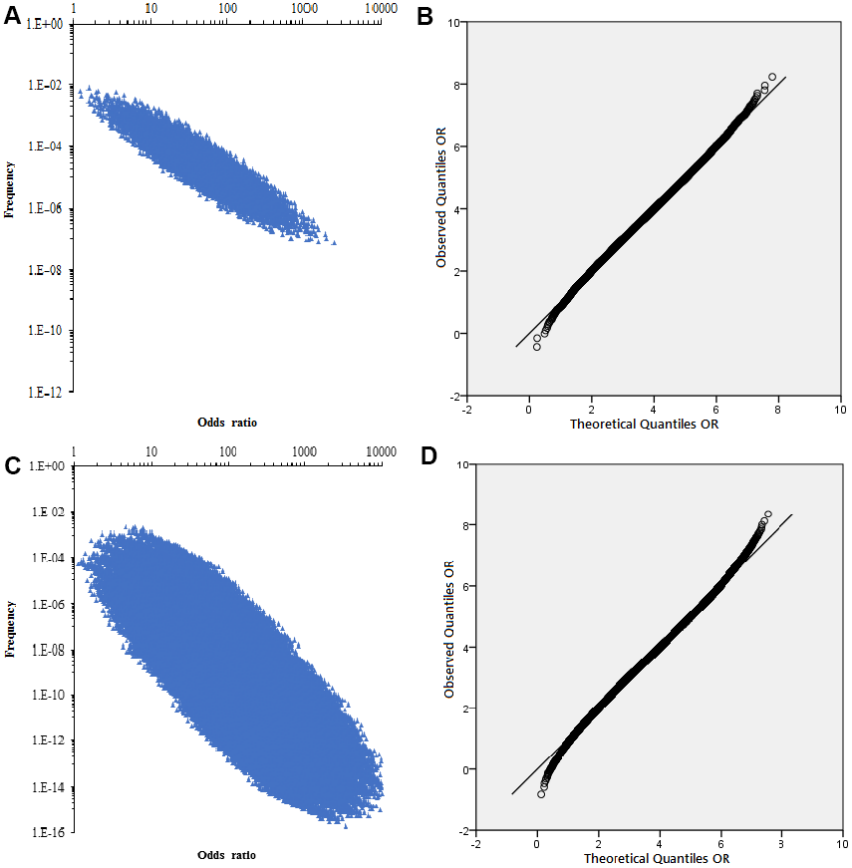
**The combined distribution of the correlation strength and Q-Q normal distribution plot.** (**A**) The combined distribution of the correlation strength (*OR*) of the 13 risk factors and the accumulation frequency. (**B**) Q-Q normal distribution plot of non-genetic factor accumulation frequency *OR*. (**C**) The combined distribution of the correlation strength (OR) of the 22 SNPs and the accumulation frequency. (**D**) Q-Q normal distribution plot of genetic factor accumulation frequency *OR*.

The combined distribution of the correlation strength (*OR*) of the 13 risk factors (*OR*>1.20) and the accumulation frequency of the risk factors was shown in Figure 5A, a total of 8,192 combinations were generated by random combination. Q-Q plot showed that the two data sets were from a population with common distribution, which conforms to normal distribution (Figure 5B).

The association strength (*OR*) of 22 SNPs with gastric cancer and the average risk of population were shown in Table 3, a total of 4,194,304 combinations were generated by random combination. The relationship between the combined *OR* and frequency of SNP was shown in Figure 5C, and each genotype exists as a categorical variable in the population. Thus, each possible combination can be represented in a 22-dimensional form, such as{XXXXXXXXXXXXXXXXXXXXXX}, representing a combination of genotypes containing all pathogenicity. Obviously, the cumulative frequency of gastric cancer risk was highly correlated with the combined *OR* value (Figure 5C). After logarithmic transformation, Q-Q plot showed that the *OR* value corresponding to the accumulation frequency conforms to the normal distribution Figure 5D.

Based on the above signifcant associations, Attributable Risk Percentage (*ARP*) and Population Attributable Risk Percentage (*PARP*) for non-genetic factors and 22 SNPs were calculated and presented in Tables 4, 5. For non-genetic factors, the top three for *ARP* were 65.87% (stomach disease),54.75% (pickled food), and 49.75% (smoked and frying). For *PARP* were 34.24% (edible hot food), 34.22% (pickled food) and 23.66% (*H. pylori* infection). On the basis of *ARP* and *PARP* associated with SNPs of gastric cancer, the top three for *ARP* were 53.91% (*NAT2*, rs1799929), 53.05% (*NAT2* phenotype), and 42.85% (*IL-10*, rs1800896). For *PARP* (Chinese Han in Beijing, CHB) were 36.96% (VDR, rs731236), 25.58% (*TGFBR2*, rs3773651) and 20.56%(*MUC1*, rs4072037).

**Table 4:** The relationship between non-genetic factors and epidemiological effect estimation for gastric cancer.

**Risk factors**	**s*OR*(*95%CI*)**	**Risk frequency**	**Epidemiological effect estimation**	
			***ARP*(%)**	***PARP*(%)**
*Hp*-infection	1.62(1.47,1.79)	0.50	38.27	23.66
Smoking	1.28(1.22,1.34)	0.42	21.88	10.52
Drinking	1.29(1.23,1.37)	0.33	22.48	8.73
Family history	1.87(1.81,1.93)	0.15	46.52	11.54
Stomach disease	2.93(2.32,2.71)	0.14	65.87	21.27
High salt diet	1.74(1.64,1.86)	0.39	43.53	22.40
Pickled food	2.21(2.03,2.38)	0.43	54.75	34.22
Fast eating	1.83(1.71,2.01)	0.39	45.36	24.25
Irregular meals	1.71(1.62,1.91)	0.32	41.52	18.51
Edible hot food	2.37(1.66,2.81)	0.38	57.81	34.24
Smoked and frying	1.99(1.46,2.70)	0.21	49.75	17.21
Spicy diet	1.73(1.16,2.63)	0.23	42.20	14.38
Mental depression	1.82(1.34,2.46)	0.22	45.05	15.28
Diabetes	0.76(0.68,0.84)	0.13	31.58	-3.22

*ARP*, attributable risk percentage; *PARP*, population attribute risk percentage.

*AR*P =|*OR*−1/*OR*|×100%; *PAR*P =|*P*e(*OR*−1)/[*P*e(*OR*−1)+1]|×100%, *P*_e_ was the mutation proportion of the control group risk percentage.

**Table 5:** The relationship between genetic variants and epidemiological effect estimation for gastric cancer.

**Gene function**	**Gene (Var.)**	**Risk allele**	**Risk allele frequency**		**sOR**	**Genetic** **score**	**Epidemiological effect estimation**		
			**Control**	**CHB**			***ARP*(%)**	***PARP*^Control^(%)**	***PARP*^CHB^(%)**
Inflammation	COX-2 (rs20417)	C	0.04	0.05	1.48	1.05	32.43	2.08	2.50
	IL-17A (rs2275913)	A	0.39	0.49	1.28	1.29	21.88	9.76	12.07
	IL-17A (rs8193036)	C	0.42	0.31	1.13	1.08	11.50	5.21	3.88
	TGFBR2 (rs3773651)	G	0.78	0.86	1.4	1.81	28.57	23.83	25.58
	IL-10 (rs1800896)	G	0.06	0.03	1.75	1.05	42.86	4.06	2.49
	VDR (rs731236)	T	0.94	0.96	1.61	2.52	37.89	36.35	36.96
Metabolism	MTHFR (rs1801133)	T	0.41	0.47	1.19	1.18	15.97	7.22	8.13
	NAT2 (rs1799930)	A	0.17	0.20	1.43	1.18	30.07	6.85	7.88
	NAT2 (rs1799929)	T	0.12	0.00	2.17	1.01	53.92	12.23	0.34
	NAT2 phenotype	Slow	0.08	0.08	2.13	1.20	53.05	8.70	8.70
	GSTM1	Null	0.49	0.49	1.32	1.34	24.24	13.51	13.51
	GSTT1	Null	0.48	0.48	1.25	1.25	20.00	10.64	10.64
Signal transduction	PLCE1 (rs2274223)	A	0.23	0.19	1.3	1.12	23.08	6.35	5.37
	PLCE1 (rs3765524)	T	0.21	0.19	1.31	1.12	23.66	6.01	5.54
	PRKAA1 (rs13361707)	C	0.48	0.50	1.42	1.46	29.58	16.69	17.21
Apoptosis/Proliferation	PSCA (rs2294008)	C	0.28	0.25	1.19	1.10	15.97	4.99	4.49
	PSCA (rs2976392)	A	0.28	0.24	1.13	1.06	11.50	3.55	3.06
	MUC1 (rs4072037)	A	0.82	0.84	1.31	1.58	23.66	20.36	20.56
Excision repair	XRCC1 (rs1799782)	T	0.28	0.29	1.4	1.24	28.57	9.95	10.28
	XRCC1 (rs25487)	A	0.29	0.25	1.11	1.06	9.91	3.11	2.70
	XRCC3 (rs861539)	T	0.14	0.07	1.29	1.04	22.48	3.92	1.93
	ERCC5 (rs751402)	A	0.33	0.35	1.21	1.15	17.36	6.39	6.93

CHB Han Chinese in Beijing, China; *ARP*, attributable risk percentage; *PARP*, population attribute risk percentage; *AR*P =|OR−1/OR|×100%.

*PAR*P =| *P*e(OR−1)/[*P*e(OR−1)+1] |×100%, *P*_e_ was the mutation proportion of the control group risk percentage.

Genetic score = (1-p)^2^+2p(1-p)OR+p^2^OR^2^, *p* was the risk allele frequency.

## DISCUSSION

### Non-genetic factors

### H. pylori infection

The prevalence of *H. pylori* infection has declined globally, however, the prevalence of gastric cancer remains high in many subgroups and geographic regions [16]. Numerous studies have confirmed that *H. pylori* is an undisputed cause of gastric cancer [17]. It is considered to be one of the major risk factors for gastric cancer [18, 19]. *H. pylori* is estimated that *H. pylori* infection accounts for 65% to 80% of gastric cancer, and about 660,000 new cases of gastric cancer occur worldwide each year [20, 21]. The main risk factor for intestinal metaplasia, chronic atrophic gastritis and gastric adenocarcinoma (GCA) is *H. pylori*, which colonizes the human stomach [22]. Studies in Asian countries have shown a high positive correlation between *H. pylori* infection and GCA, while other studies in Western countries have not found a correlation or even a reverse association [23, 24].

In this study, we provided evidence from 119,117 Chinese participants for a positive association between gastric cancer and *H. pylori*. For gastric cancer, summary *OR* was 1.62 (95% CI: 1.47-1.79), greater in cardiac gastric cancer (*OR* = 1.80; *95% CI* 1.55-2.09) than in non-cardiac gastric cancer (*OR* = 1.46; *95% CI*: 1.24-1.72). The analysis of time trend of *H. pylori* infection was consistent with the latest systematic review and meta-analysis [25]. However, it is also estimated that 89% of non-cardia gastric cancer cases are due to this infection in American [26]. This may be related to the genetic background and geographic area of the ethnicity.

### Family history

Family history is a definite and strong risk factor for gastric cancer [27, 28]. Although most gastric cancers are sporadic, about 10% of gastric cancers have family aggregation [29]. The development of gastric cancer under the age of 50 may be accompanied by a family history [30]. The history of the gastric cancer family has increased the risk of its development, with the risk of first-degree relatives of gastric cancer cases ranging from 1.3 to 3.0, suggesting family history of gastric cancer is an independent risk factor [31, 32].

The evidence of family history as a risk factor for gastric cancer has been evaluated in the present study, we combined 108 studies on the association between gastric cancer and family history in China, the results showed that the family history of gastric cancer was significantly associated with gastric cancer (*OR* = 1.87; *95% CI*: 1.81-1.93), non-cardiac gastric cancer (*OR* = 3.28; *95% CI*: 1.75-6.13) and cardiac cancer (*OR* = 2.46; *95% CI*: 1.25-4.85), respectively. Therefore, determining genetic parameters of subjects with a family history of gastric cancer is an important step in the correct diagnosis and treatment of the disease.

### Smoking and drinking

Numerous studies have shown that the relationship between smoking and gastric cancer is not sufficient to include smoking as a risk factor for gastric cancer, but the International Agency for Research on Cancer (IARC) concluded in 2002 that there is sufficient evidence to support smoking as a risk factor for cancer [12]. This is consistent with our findings from 286 Chinese studies, the smoking was an independent risk factor for gastric cancer (*OR* = 1.28; *95% CI*: 1.22-1.34), non-cardiac gastric cancer (*OR* = 1.24; 95% CI: 1.12-1.37) and cardiac cancer (*OR* = 1.33; *95% CI*: 1.12-1.57), respectively.

Both tobacco smoke and alcohol are established carcinogens, alcohol consumption is considered a typical carcinogenic factor in the latest list of carcinogens published by the IARC [33]. Epidemiological studies have consistently argued that high alcohol consumption is associated with an increased risk of gastric cancer and reports a strong dose-response relationships [34]. In our study, we analyzed the relationship between alcohol consumption and gastric cancer from 211,079 participants. The results indicated that alcohol consumption was positively associated with gastric cancer risk (*OR* = 1.29; *95% CI*: 1.23-1.37), non-cardiac gastric cancer (*OR* = 1.39; *95% CI*: 1.09-1.77) and cardiac cancer (*OR* = 1.45; *95% CI*: 1.29-1.64), respectively.

### Stomach disease

Previous studies have shown that peptic ulcer disease, stomach and duodenal diseases have emerged or become more prevalent in Western countries in the 19th century, probably due to changes in the epidemiology of *H. pylori* infection [35]. The risk of gastric cardia cancer is related to gastroesophageal reflux disease [12, 36]. However, the evidence does not indicate that these risk factors are associated with non-cardia cancer [37, 38]. Our study suggested that the history of gastric diseases was strongly associated with gastric cancer (*OR* = 2.9, *95% CI*: 2.32-2.71) in Chinese population.

### Diet

Studies have shown that smoked meat and salt-preserved foods can cause cancer, and large intakes increase the risk of *H. pylori* infection [39, 40]. Bacon produces N-nitroso compounds associated with gastric cancer, and many epidemiological and experimental studies support this hypothesis [41, 42], including our findings (*OR* = 1.99; *95% CI*: 1.46-2.70 for smoked or frying meat, *OR* = 2.21, *95% CI*: 2.03-2.38 for pickled food).

A large cohort study in South Korea showed that people who eat more salty foods have a higher risk of gastric cancer, because salty foods can directly damage the gastric mucosa and cause gastritis [16]. This is consistent with the results of our research (*OR* = 1.74, *95% CI*: 1.64-1.86).

Bulgarian researchers have found that during the economic crisis, radiologically recorded gastroduodenal ulcers have increased, and they believe that no meals and continuous smoking are the causes of ulcers [43]. The recent studies showed that frequent deviation in meal timing over a prolonged period appears associated with increased risk of *H. pylori* infection and gastritis [44]. Based on these considerations, we conducted a quantitative analysis of the relationship between irregular diet and gastric cancer in China. The results showed that both irregular diet and fast diet were independent risk factors for gastric cancer (*OR* = 1.71, *95% CI*: 1.62-1.91 for irregular meals and *OR* = 1.83, *95% CI*: 1.71-2.01 for fast eating).

An epidemiological survey in India shows that spicy diet is an independent risk factor for gastric cancer [45]. The impact of dietary habits on gastric cancer showed that regular consumption of fried or grilled food and consumption of spicy food were important factors associated with gastric cancer in males and females [46]. In Chinese population, our research further confirmed that spicy diet (*OR* = 1.73, 95% CI: 1.16-2.63) and edible hot food (*OR* = 2.37, 95% CI: 1.66-2.81) are significantly associated with gastric cancer. According to the above epidemiological evidence, dietary habit was an important factor contributing to gastric cancer.

### Mental depression

Studies have shown that leptin and its receptor (leprb) are independent factors for the poor prognosis of patients with gastric cancer and suggest that leptin-leperbis is a necessary factor for the action of antidepressants [47]. Depression is a common symptom in patients with gastric cancer and a potential marker for poor prognosis and clinical stage of advanced cancer [48]. The study found Leptin-leprb plays an important role in the pathogenesis and depression of gastric cancer Leptin-leprb may be a potential diagnostic marker and therapeutic target for gastric cancer. We found a significant statistical association between mental depression and gastric cancer through quantitative combine of observational epidemiological studies (*OR* = 1.82, *95% CI*: 1.34-2.46).

### Other factors

The risk of cardia cancer is related to obesity and gastroesophageal reflux disease [36]. Epidemiological and evidence-based medicine studies have similar results. People with BMI than 30 kg/m^2^ have a significantly increased risk of cardiac cancer [49, 50]. However, we did not find the association between gastric cancer and obesity (*OR* = 0.79, *95% CI*: 0.53-1.21). Meanwhile, we did not find a statistically significant association between hypertension and gastric cancer (*OR* = 0.95, *95% CI*: 0.88-1.01). It is worth noting that we found that the risk of gastric cancer was lower in the diabetic population than in the general population (*OR* = 0.76, *95% CI*: 0.68-0.84). This may be due to the change of diet structure and living habits in patients with diabetes mellitus, and it is also related to taking metformin [51].

### Genetic risk factors

A considerable number of patients with gastric cancer have potential genetic predisposition syndromes, especially those with a family history of early gastric cancer and other cancers [52]. Once an individual is found to have a predisposition to gastric cancer, screening or risk reduction procedure can be initiated to detect or prevent cancer early [53]. Therefore, identifying risk-related biomarkers is essential for early detection of gastric cancer. In this study, we assessed the credibility and intensity of a significant association between candidate gene SNPs and GC risk, providing comprehensive information for further research.

### PLCE1 rs2274223 and rs3765524

Phospholipase C epsilon 1 (PLCE1) plays an important role in cell growth, differentiation and carcinogenesis. The rs2274223, located in exon 26 of the *PLCE1*, is a non-synonymous SNP that causes an amino acid to change from histidine to arginine [54]. An increasing amount of studies have begun to explore the relationship between *PLCE1* rs2274223 polymorphism and susceptibility of different cancers [55]. In this study, a statistically significant association was observed in gastric cancer for rs2274233 (A vs. G: *OR* = 1.30; *95% CI*: 1.23-1.37). This is consistent with the results of large sample published studies [55, 56]. The rs3765524 is in exon 24 and in Y domain. GWAS found that SNPs in the PLCE1 are mainly rs2274223 A>G and rs3765524 C>T, which are common susceptibility sites for esophageal cancer and gastric cancer [14, 54, 57, 58]. Because they are completely linkage disequilibrium (LD) (r^2^=1.00) (http://grch37.ensembl.org/Homo_sapiens/Tools/LD/Results?tl=YduaFHeCYXzsiKQX-5544662), the results were basically the same (A vs. G: *OR* = 1.30; *95% CI*: 1.22-1.38), and they were all the SNPs of *PLCE1* in Chinese population [59].

### PSCA rs2976392 and rs2294008

The prostate stem cell antigen (PSCA) gene is located on chromosome 8q24.2 and encodes a 123-amino acid cell surface protein with 30% homology to type 2 stem cell antigen (SCA-2) [60]. The *PSCA* polymorphism is associated with high expression of PSCA in cancer patients [61]. The T allele of *PSCA* rs2294008 could decrease the transcriptional activity of the *PSCA* promoter in gastric cell lines [62]. The rs2976392 G>A and rs2294008 C>T polymorphisms are the most widely studied polymorphisms in the *PSCA*, and have been proved to be associated with an increased risk of gastric cancer [63]. LD analysis for *PSCA* rs2294008 and rs2976392 in Chinese Han population indicated that they have a strong LD (r^2^=0.974) (http://grch37.ensembl.org/Homo_sapiens/Tools/LD/Results?tl=7sSPOBfXyLzee3pP-5720247). The online LD value prediction result is consistent with the published study [63]. Our findings also confirm this association (rs2976392: C vs. T: OR = 1.13, 95% CI: 1.05-1.22; rs2294008: A vs. G: *OR* = 1.19, *95% CI*: 1.12-1.27).

### MUC1 rs4072037

Abnet et al. conducted a GWAS of the Chinese population in 2010 and found that the *MUC1* rs4072037 polymorphism is related to gastric cancer risk [14]. In 2011, Saeki et al. performed a GWAS of gastric cancer in the Japanese population and found that the *MUC1* rs4072037 polymorphism gastric cancer risk [57]. In this study, the A allele was significantly associated with an increased risk of gastric cancer (A vs. G: *OR* = 1.31, *95% CI*: 1.21-1.43). This is close to the GWAS result.

### PRKAA1 rs13361707

The 5'-AMP activated protein kinase (AMPK) is encoded by the AMP-activated protein kinase catalytic subunit alpha-1 gene (*PRKAA1*) located on chromosome 5p13.1 [64]. The *PRKAA1* rs13361707 T>C is the most widely studied polymorphism associated with gastric cancer risk. Previous GWASs found that the *PRKAA1* rs13361707 was a risk factor for non-cardiac gastric cancer in Chinese populations [15]. However, these results were not successfully replicated. In the present study, a significant relationship was revealed between the *PRKAA1* rs13361707 T>C and gastric cancer susceptibility under all genetic model (C vs. T: *OR* = 1.42, *95% CI*: 1.35-1.48, CT vs. TT: *OR* = 1.50, *95% CI*: 1.37-1.63; CC vs. TT: *OR* = 2.03, *95% CI*: 1.84-2.23; CT+CC vs. TT: *OR* = 1.66, *95% CI*: 1.53-1.80; CC vs. CT+TT: *OR* = 1.53, *95% CI*: 1.42-1.66). The results are consistent with those of Jiang et al. [65].

### Other significant associations

Among 22 significantly associated SNPs, the level of evidence for high and intermediate were 3 (13.64%) and 19 (86.36%), respectively. The above discussion mainly involved SNPs with high quality evaluation and their LD SNPs or discovered by GWAS. The remaining SNPs (*IL-17A*, rs2275913, rs8193036; *IL-10*, rs1800896; *MTHFR*, rs1801133; *COX-2*, rs20417; *XRCC1*, rs1799782, rs25487, rs861539; *NAT2*, rs1799929, rs1799930, phenotype; *GSTM1*; *GSTT1*; *ERCC5*, rs751402; *TGFBR2*, rs3773651; *VDR*, rs731236) are also significantly associated with the risk of gastric cancer.

### Epidemiological evaluation of risk factors

In this study, we firstly analyzed 13 non-genetic factors risk and 22 common SNPs as each independent unit using a programming program, and analyzed the distribution of *OR* values corresponding to each accumulation frequency. The *ORs* value corresponding to the accumulation frequency are in accordance with the normal distribution, and the *ORs* value gradually increases as the accumulation frequency decreases.

The *ARP* and *PARP* indicated the number of gastric cancers among exposed individuals that can be attributed to that exposure. Among the controllable factors, changing unreasonable eating habits and bad behaviors, it can effectively reduce the risk of gastric cancer, and has good socioeconomic benefits. On the basis of *ARP* and *PARP* associated with SNPs of gastric cancer, we calculated the frequency of allele variation in the control and Chinese population, respectively, and the results were consistent. In the present study, we firstly presented the data on *ARP* and *PARP* for effects of total gastric cancer-related SNPs based on the data from public databases and human genome (HapMap) project to evaluate the contribution of SNPs to the occurrence of gastric cancer.

There are some limitations should be pointed out. Among the included studies, the proportion of cohort studies was relatively low. In addition, the heterogeneity of some risk factors was high after combined analysis, which reduces the credibility of the results to a certain extent. Further, some non-genetic factors cannot be performed due to insufficient data in the stratification analysis of gastric cancer subtypes. Finally, the interaction between independent risk factors was not addressed, such as the interaction between environmental risk factors and environmental or genetic factors, and the interaction between SNP and SNP were ignored in the analysis of multiple gene association, interactive and additive effects are recommended for exploration in future studies.

## CONCLUSIONS

In summary, we conducted the first field synopsis in Chinese population linking common non-genetic risk factors and DNA variation to summarize potential non-genetic and genetic risk factors for gastric cancer, and to evaluate their epidemiological significance. We hope that comprehensive data collection will provide a useful platform for researchers who may be responsible for the pathogenesis of gastric cancer.

## MATERIALS AND METHODS

This study was conducted based on the Meta-analysis of Observational Studies in Epidemiology (MOOSE) [66], Preferred Reporting Items for Systematic Reviews and Meta-Analysis (PRISMA) guidelines [67] and the systematic review principles of molecular association studies proposed by the Human Genome Epidemiology Network (HuGENet) [68–70].

### Search strategy

A systematic literature searching was implemented using PubMed, EMBASE, Cochrane Library, Web of Science, CNKI (Chinese), Wanfang (Chinese), VIP (Chinese) and CBM (Chinese) database updated to November 28, 2020. The combination terms for retrieval were keywords: “risk factor” and “*H. pylori* infection” and “*H. pylori* colonization”; “gastric” and “stomach” or “junctional and non-junctional”; “carcinoma”, “tumor” and “cancer”; “polymorphism”, “single nucleotide polymorphism” and its acronym “SNP”, “variant”, “variation”; “China” and “Chinese” and “Chinese Population”. To further identify potential articles, we also manually retrieved bibliography of relevant studies that were not retrieved by literature databases, and HuGENet Phenopedia, http://www.hugenavigator.net/HuGENavigator/startPage PhenoPedia was searched.

### Inclusion and exclusion criteria

The inclusion criteria were as follows: (i) about the polymorphisms or non-genetic factors and gastric cancer risk in Chinese populations; (ii) case–control or cohort-designed study; (iii) studies with raw data or summarizing data for estimating an odds ratio (OR) with 95 % confidence interval (CI); (iv) and (4) available genotype frequencies. Exclusion criteria were data published only in abstract form, with minor allele frequencies <1% (rare variation) in the control group, sample size <10 cases or control group, and the genotypes of controls were not in accordance with the assumptions of HWE.

### Data extraction

Data extraction was conducted independently by two authors (FJD and CHS) and discrepancies were finalized after consultation with third author (KJW). The information extracted from included studies if available: the first author’s name, publication year, sample size, nations, demographics, gastric cancer subtype (gastric cancer, non-cardiac gastric cancer and cardiac cancer) and Lauren classification (intestinal, diffuse and mixed type), design of study, genotype distributions and potential risk factors (*H. pylori* infection, smoking, drinking, family history, stomach disease, high salt diet, pickled food, fast eating, irregular meals, edible hot food, smoked and frying, spicy diet, mental depression, body mass index (BMI), hypertension, diabetes). If duplicate publications using the same population were found, data from the most recent publication were included.

### Assessment of cumulative evidence

To assess the credibility of each nominally statistically significant association determined by pooled analysis, we adopted the Venice standard [68, 71–73]. In short, the level of confidence is defined by the level of three parameters (A = strong, B = medium or C = weak), the grades may be scored as follows: ①AAA– strong evidence. ②AAB, ABA, ABB, BAA, BBA, BBB, BAB– moderate evidence. ③Rest all scores will be treated as poor, unreliable evidence. In addition to the Venice standard, we also assessed the significance findings by calculating the false positive reporting probability (FPRP) [74, 75]. In the early stage, statistically significant genetic association studies, even with large sample sizes, were well implemented, avoiding all research biases, and the probability of false positives was still high. Studies have shown that at least 95% of the research results are statistically significant, which we call "no real correlation probability", which is FPRP.

### Statistical analysis

The pooled *ORs* with *95% CIs* were performed by Review Manager 5.3.5 (Cochrane Collaboration, Oxford, UK) to evaluate relationship between genetic variant, potential risk factors and gastric cancer risk. For some studies, including GWAS, only per-allele *OR* were available, and genetic modeling is carried out by calculating an allele model. For each variant or potential risk factor, a meta-analysis was performed if at least three data sets were available. As for GWAS, the discovery and verification phases are treated as separate data sets.

Meta-analysis used a fixed effect model (*P*-value of heterogeneity (*P*_heterogeneity_) ≥ 0.10 or *I*^2^ ≤ 50%) or a random effects model (*P*_heterogeneity_ < 0.1 and *I*^2^ > 50%) based on the inter-study heterogeneity, and we obtained the distribution of observed and expected values by quantile-quantile (Q-Q) plots. The trend distributions were performed with Visual Studio 2013 (Microsoft Corporation, Redmond, USA) to explore by possible combinations and frequencies of genetic variants and potential risk factors.

The relative risk (*RR*) of the exposed part of the population is divided into the *RR* of the unexposed part of the population, and the relative measure of the given exposure effect (*RR*) is obtained. If the occurrence is rare, it is approximately *RR* (*RR*≈*OR*), the RR was estimated using the summary estimates (*OR*) calculated by the meta-analysis.

*ARP*- and *PARP* as indexes were used to assess the effect of epidemiology.

*ARP* = | *OR*−1/*OR*|×100%

*PARP* =*P*_e_| (*OR*−1)/[*P*_e_(*OR*−1)+1] |×100%

*P*_e_ was the mutation proportion of the control group.

The population average risk (Genetic score) of single SNP was calculated based on the genotype frequency of the genetic variation in the haplotype map of the HapMap and the pooled *OR* of the meta-analysis in the Chinese population.

Genetic score = (1-*p*)^2^+2*p*(1-*p*)*OR*+*p*^2^*OR*^2^

*p* was the risk allele frequency.

The Q-Q plot is a graphical technique for determining if two data sets come from populations with a common distribution. All *P*-values were two-sided and *P* < 0.05 was considered statistically significant. The sensitivity analysis and publication bias were performed using STATA 13.1 (StataCorp, College Station, TX, USA).

## Supplementary Material

Supplementary References
