# Supplementary material for: Identification and epidemiological evaluation of gastric cancer risk factors: based on a field synopsis and meta-analysis in Chinese population
Source: Aging (Albany NY). 2021 Sep 6;13(17):21451–69. doi: 10.18632/aging.203484 (PMC8457565; doi:10.18632/aging.203484)
Supplement: Supplementary References [file aging-13-203484-s001.pdf]

SUPPLEMENTARY REFERENCES

1. Ayinuer Aheman, Meilikezati Anzaer, Lizha Juma, et al. Correlation of the expression of Helicobacter pylori CagA and VacA with gastric cancer risk in Xinjiang Uygur population[J]. Hainan Medical Journal, 2017(19).

2. BAI Xue-Lei, SUN Li-Ping, LIU Jin, et al. Correlation of Interleukin-10-1082G /A Single Nucleotide Polymorphism to the Risk of Gastric Cancer in North China: A Case-Control Study[J]. Chinese Journal of Cancer, 2008, 27(1) : 35- 40

3. Bao PP, Gao LF, Liu DK, et al. A Case-Control Study of the Relationship between Diet and Stomach Cancer in Shanghai Resident[J]. China Oncology, 2003,12(2): 66-70

4. Bi JP. MTHFR C677T and P53 Condon 72 Pro/Arg Polymorphisms and Susceptibilities to Cardia and Non-cardia Gastric cancer[D]. Fujian Medical University, 2005.

5. Cao CL. Multivariate analysis of the correlation between IL-6 174G/C gene polymorphism and susceptibility of stomach cancer[J]. Modern Preventive Medicine, 2014(20).

6. Zeng HM, Pan KF, Zang Y, et al. Relationship between toll-like receptor 2 and toll-like receptor 9 gene polymorphism and gastric cancer susceptibility[J]. China Preventive Medicine,2011,45(7):588-592.

7. Chai ZZ, Yao LQ, Wang JE, et al. Study on the relationship between type 2 diabetes mellitus and gastric cancer[J]. China Modern Doctor, 2018(23):102-105.

8. Jv JH. A 4bp Insertion/deletion Polymorphism within RERT-lncRNA and the Susceptibility to Multipe Cancers[D]. Soochow University, 2013,1-23.

9. Cen DJ. Interaction between the genetic polymorphism of NQO1,XRCC1 and environmental exposure and their risks for Gastric cancer[D]. Anhui Medical University, 2007.

10. Chen FG, Zheng ZR, Wu XQ, et al. Association of Prostate Stem Cell Antigen Gene rs2294008 Polymorphism with Gastric Cancer in Chinese Han Population[J]. Gastroenterol, 2010(01):29-32.

11. Chen H, et al. PLCE1 gene and the risk of Qinghai hui gastric cancer in the related research[D].2016.

12. Chen JS, Chen ZC, Chen XC, et al. Dietary and Other Living Habits and the Risks of Gastric Cancer in Change, a High-Risk Area in China[J]. Chinese Journal of Viral Diseases, 2002,4(3):131-134.

13. Chen J, Qi F, Huang Y, et al. The study on the relationship between polymorphism of MMP-9 and gastric cancer[J]. Modern Preventive Medicine, 2013(12):151-155.

14. Chen JJ. Association Study of Polymorphisms in IL23R and IL17A Genes with the Susceptibility of Gastric Cancer[D]. Nanjing Medical University, 2010.

15. Chen JL, Wen Y, Chang H, et al. The correlation between IL-1B and IL-1RN gene polymorphism and risk of non-cardia gastric cancer[J]. Modern Preventive Medicine, 2016, 43(2).

16. Chen L. Genetic polymorphisms and disease risks:STK15gene and risk for gastric cancer, MMP9 gene and risk for Thoracic Aortic Aneurysm and Thoracic Aortic Dissection[D]. China Medical University, 2005.

17. Chen LS. Significance of Expression and SNPs of MTDH in Gastric Cancer[D]. Fujian Medical University, 2011.

18. Chen P， Wu CC， Chen Y, et al. Influencing factors of gastric cancer in xianyou， Fujian[J]. Modern Preventive Medicine, 2018, 45(19):104-108.

19. Chen SQ. Relationship between single nucleotide polymorphism of TRIT1, MYCL1 and MFSD2A gene region and the development of gastric cancer[D]. Fujian Medical University, 2011.

20. Chen SQ, Hong BB, Lin XD, et al. Association of the e NOS G894T polymorphism with the development and progression of gastric cancer[J]. Journal of Chongqing Medical University, 2010, 35(12):1816-1819.

21. Wang Y, Yu LP， Zhang FJ, et al. Correlations of MMP-2 and TIMP-2 polymorphisms with the risk of gastric cancer[J]. J Dig Oncol（Electronic Version, 2015, 7(3):143-149.

22. Chen WS.A molecular epidemiological study on the association between functional polymorphisms of IGFBP3 and the susceptibility to gastric cancer[D]. Nanjing Medical University, 2008.

23. Chen WS. Igfbp-3 gene polymorphism and genetic susceptibility to gastric cancer: a case-control study [C]// national symposium for graduate students in environmental and occupational medicine. 2006.

24. Chen XL. Associations of IL-1related SNPs with gastric cancer risk and functional verification[D]. ZhengZhou University, 2016.

25. Chen X. Study on the correlation between micro-RNA single nucleotide polymorphism and the characteristics of gastric cancer incidence and prognosis[D], China Medical University, 2017.

26. Chen Y. Association Analysis of SNPs between lncRNA colon cancer associated transcript-1 SNP and Gastric Cancer Susceptibility[D]. ZhengZhou University, 2017.

27. Cheng JG, Zhu C, Du JB, et al. Nonsynonymous polymorphisms in FAT4 gene are associated with the risk of gastric cancer in a Jiangsu population[J]. Journal of Nanjing Medical University (Natural Sciences), 2013(11):1638-1642.

28. Cong L. Mechanism of vitamin D receptor modulating WNT/β-catenin signaling pathway in the proliferation and invasion of gastric cancer[D]. SanDong University, 2016.

29. Dai NB. Association Study of Genetic Variants at 5p13.1 with Non-cardia Gastric Cancer[D]. Nanjing Medical University, 2015.

30. Deng XL, Chen M, Chen W, et al. Relationship between DNA repair gene polymorphism and gastric cancer susceptibility[J]. Journal of Molecular Diagnostics and Therapy, 2010, 02(6):371-374.

31. DI Ji，ZHAO Jun—hui. Risk of Gastric Cancer and Polymorphism of CYP2E1 Gene in Tibetan and Hui at Qinghai[J]. Chinese High Altitude Medicine and Biology, 2017(2):109-116.

32. Ding JH , Cao HX , Li SP , et a l. Relationship of ADH2, ALDH2 Genotypes and Alcohol Consuming with Risk of St om ach Cancer[J]. China Cancer, 2011, 20(8):579-583.

33.Ding JH, Wu JZ, Gao CM, et al. The relationship between genetic polymorphism of aldehyde dehydrogenase-2 and alcohol drinking for the susceptibility of the primary hepatocellular carcinoma[J]. Cancer, 2004, 24(4):450-452.

34. Dong X. Study on the correlation between polymorphism of MiR-107 gene and genetic susceptibility to gastric cancer[D]. JiangSu University, 2012.

35. Duan ZP. Functional polymorphism of ERCC5 promoter and its interaction with environmental factors and risk of gastric cancer[D]. 2013. 36.

Er LJ. The relationship between MDM2 gene polymorphism and esophageal, gastric and double primary cancer[D]. 2010.

37. Fang Q, Yang GF. The Correlation Analysis of Cytokine Gene Expression and Gastric Cancer[J]. Chinese Journal of Medicinal Guide, 2012(10).

38. Fei SJ, Xiao SD. Non- dietary factors and gastric cancer: a case- control study[J]. Acta Academiae Cademice Medicinae XuZhou, 2004(05):5-9.

39. Feng JB, Nie YQ, Lin Y, et al. Association between single nucleotide polymorphism at miR-191 target site and susceptibility to gastric cancer[J]. The Journal of Practical Medicine, 2014, 30(8):1217-1220.

40. Feng YX. Family Comparative Study of Inflammatory Cytokine Gene Polymorphisms and Gastric Cancer Susceptibility[D]. ZhengZhou University, 2008.

41. Fu G, Yang YF, Cheng XB, et al. Relationship between the Methylenetetrahydrofolate Reductase A1298C Genetic Polymorphisms and Susceptibility to Gastric Cancer[J]. J Environ Occupational Med, 2007(01):36-39.

42. Gao LJ. The correlation between interleukin-10 gene polymorphism and gastric cancer[D]. 2013.

43. Gao LJ, Song CH, Li HX, et al. Inflammatory cytokine gene-environment interaction on risk of gastric cancer susceptibility[J]. Chin J Public Health, 2013, 29(6): 799-801.

44. Gao RP, Yang L. A case-control study of 208 cases of gastric cancer risk factors in NingXia[J]. Journal of Ningxia Medical University, 2011, 33(12):1170-1174.

45. Gao Y, Cui YX, Jiang XJ. Association of Dnmtl single-nucleotide polymorphisms and risk of gastric cancer[J]. Chinese Journal of Cancer Prevention and Treatment, 2015, 7(06):12-15.

46. Gao CM, Sugimura, Haruhiko, et al.hOGG1 Genotypes, Life Style and the Risk of Esophageal and Stomach Cancers[J]. China Cancer, 2001(9):500-502.

47. Gao CM, TakezakiToshiro, Wu JZ, et al. Effects of GSTT1 and GSTM1 Genotypes, Lifestyle Factors and Their Interactions on Risk of Esophageal and Stomach Cancers[J].Chinese Journal of Cancer Prevention and Treatment, 2002(2).

48. Gao CM, Wu JZ, Ding JH, et al. Polymorphisms of methylenetetrahydrofolate reductase C677T and the risk of stomach cancer[J]. Chinese journal of Epidemiology, 2002, 23(4):289-292.

49. Gao CM, Wu JZ, Liu YT, et al. Interactions between lifestyle, methylanetetrahydrofolate reductase gene and polymorphisms in thymidylate synthase gene with risk of stomach cancer[J]. Chinese journal of Epidemiology, 2003, 24(7):599-603.

50. Gong YT. Effect of DNA repair gene ERCC4 polymorphism on risk of atrophic gastritis and gastric cancer[D]. 2012.

51. Long WD. The role of miR-196a2 rs11614913 SNP in genetic susceptibility, prognosis and chemotherapy sensitivity of gastric cancer[D]. Suzhou University, 2015.

52. Gu HJ. Genetic polymorphisms of RAGE, ECE-1b and TIMP2 and susceptibility to gastric cancer[D]. Nanjing Medical University, 2009

53. Gu XP, Wang YC, Zhi HG, et al. Risk factors of esophageal and stomach cancer and their clustering in Dafeng municipality: a case-control study[J]. Chin J Public Health, 2016, 32(10):1406-1409.

54. Guo W, Chen P, Zheng LH, et al. Correlation between MTHFR gene polymorphism and gastric cancer[J]. World Chinese Journal of Digestology, 2012, 20(08): 690-693.

55. He BS. Study on the correlation between immune related gene polymorphism and preoperative inflammatory markers and gastric cancer susceptibility and prognosis[D]. Nanjing Medical University , 2017.

56. He CY. Screening of candidate tagSNP gene for gastric cancer and its risk of precancerous disease in northern China[D]. 2011.

57. He H. A case-control study on the relationship between smoking and drinking and gastric cancer[D]. 2009.

58. He J. Polymorphisms in nucleotide excision repair genes and gastric cancer risk[D]. Fudan Univesity, 2013.

59. He XA, Hao NN, Zou ML. Analysis of related factors of gastric cancer[J]. Jilin Yixue, 2014(31):7015-7017.

60. Hong BB. Association of thrombospondin-1,2 single nucleotide polymorphisms with genetic susceptibility to gastric cancer[D]. Fujian Medical University, 2010.

61. Hou RP. Association of Cytotoxic T lymphocyte-associated antigen 4 gene polymorphisms with the susceptibility to gastric cancer[D]. Shandong University, 2009.

62. Huag F. A Case-control Study of the Risk Factors of Gastric Cancer among the Uygur group and the Han group in XinJiang [D]. Xinjiang Medical University, 2010.

63. Huang GN, Diao YQ, Jiang XJ. Relationships between the rs2070803 polymorphism of the MUC1 gene and susceptibility to gastric cancer[J]. Chin J Clinicians(Electronic Edition), 2013, 7(23):35-37.

64. Huang GP, Zheng ZL, Cai L. DNA repair gene XRCC3 Thr241Met polymorphism and susceptibility to cardia and non-cardia gastric cancer: a case-control study[J]. Chinese Journal of Epidemiology 2006, 27(5):420-423.

65. Huang H. Polymorphisms of TLR4 and gastric cancer susceptibility[D]. Nanjing Medical University, 2010.

66. Huang LY, Yang L, Bai DL. Risk factors of 126 cases of gastric cancer[J]. Journal of Ningxia Medical College, 2008(1).

67. Huang XX. Polymorphisms of XPA and XRCC3, environmental Agent and Susceptibilities to Esophageal, Cardia and Non-cardiac Gastric Cancer[D]. Fujian Medical University, 2007.

68. Huang X. A case-control study on the association between genetic polymorphisms of CYP1A1, GSTM1 and gastric cancer susceptibility in GuangXi Province[D]. Guangxi Medical University, 2007.

69. Jia K, Gong J, Li YC. Relationship between vascular endothelial growth factor gene-460C / T polymorphism and non-cardiac gastric cancer[J]. Chinese Journal of Gastroenterology and Hepatology, 2012, 21(5):399-402.

70. Jia K, Gong J, Li YC, et al. Study on the relationship between polymorphism of cyclin D1 gene and gastric cancer[J]. Chinese Journal of Digestion, 2008, 28(10):690-693.

71. Jia YY. Genetic chips and magnetic separation techniques were used to study the susceptibility of single nucleotide polymorphisms (SNPS) to gastric cancer in northern Jiangsu province[D]. 2012.

72. Jiang JC. The Selection and Functional Characterization of Long Non-coding RNAs and Functional SNPs Associated with Gastric Cancer Susceptibility Based on Bio-information[D]. Zhengzhou University, 2017.

73. Jiang M, Li D, Yang LP, et al. A case — control study on the relationship between diet habit and gastric cancer in Xian ，China[J]. Modern Oncology, 2012(7):1453-1456.

74.Jiang MH. Study On The Association of Environmental Factors、COPS2、CTSF、NT5E、TERF1 MiRNA Related SNPs With Gastric Cancer Risk[D]. Fujian Medical University, 2017.

75. Jang MH, Liu BY, Wu CC, et al. 1:2 Matched Case-control Investigation on Risk Factors of Gastric Cancer in Xianyou County[J]. Cancer Research on Prevention and Treatment, 2016, 43(10): 898-902.

76. Jiang AR, Wu JZ, Gao CM, et al. Relationship between polymorphisms of methylenetetrahydrofolate reductase A1298C and the susceptibility of gastric cancer[J]. Hebei Medical Journal, 2004, 26(11):851-853.

77. Jiang YH, Ju ZY, Ren CS, et al. Study on the Relationship between the Glutathiones-transferase Gene Deletion Environmental Factors and Susceptibility to Gastric Carcinoma[J]. China Public Health, 2000, 16(10):877-879.

78. Jiang GJ, Wang HM, Zhou Y, et al. The correlation study between the nucleotide Polymorphisms of cyclooxygenase-2 gene and the susceptibility to gastric cancer[J]. Journal of Nanjing Medical University (Natural Sciences), 2007, 27(8):890-894.

79. Jiang H. A study on the association rs750064, rs17878362 with risk of gastric cancer[D]. Soochow University, 2015.

80. Jiang H, Fu L, Liao AD, et al. Association study between NF-κB1 gene polymorphism loci rs4648127 and susceptibility of gastric cancer[J]. Journal of Nantong University (Medical Sciences), 2017(37):100.

81. Jiang L, Wang J, Ren XF, et al. Relationship between CYP2E1 gene rs8192775 polymorphism and interaction of gene-environment and susceptibility of gastric cancer[J]. Journal of Southeast University (Medical Science Edition), 2013(6):728-732.

82. Jing XD. A population-based case-control family study of gastric cancer[D]. Zhengzhou University, 2006.

83. Jin GF, Wang LN, Cheng WS, et al. TGFB1 and TGFBR Ⅱ relationship between gene polymorphism and susceptibility to gastric cancer research[C]// The fifth national graduate conference on environmental and occupational medicine. 2006.

84. Jin QE, Su JR. Association of Epstein – Barr Virus A73 Gene Extron A157154C Single Nucleotide Polymorphism with Gastric Carcinoma in Han Population of Beijing Area[J]. J Mod Lab Med, 2018(3).

85. Jing JJ. Study on the risk of gastric cancer and its precancerous diseases with ERCC8 Tag SNPs[D]. 2012.

86. Ju ZY. Environmental factors and glutathione transferase (GSTM1) genotype of gastric cancer susceptibility[D]. 2001.

87. Ke Q. Molecular Epidemiology of Study on Transforming Growth Factorβ(TGFβ) Signaling Pathway and Gastric Cancer[D]. Nanjing Medical University, 2008.

88. Li C. The Association of TNF Gene Polymorphisms with Helicobacter pylori Infection in Gastroduodenal Diseases[D]. Wuhan University, 2004.

89. Li CH. Polymorphisms of DNA Repair Gene XRCC4 and Susceptibility to Gastric Cancer in Chinese[D]. Shandong University, 2009.

90. Li D. The Study On The Association Of NF-kappa B Signaling Pathway Related Genes SNPs And Environmental Inflammatory Factors With The Gastric Cancer[D]. Fujian Medical University, 2015.

91. Li D, Wu CC, Lin XS, et al. Case control study on the environmental risk factors for 335 cases of gastric cancer in Xianyou county, Fujian province[J]. Modern Preventive Medicine, 2017(02):27-31.

92. Li DP. The Association of Gastric Cancer with XPC Gene Haplotype[D]. Shandong University, 2010.

93. Li F. Association Study of Single Nucleotide Polymorphisms in PSCA, MUC1, SMAD7, TNF-α and SEPS1 Gene with Risk of Gastric Cancer in a Hunan Population[D]. Central South University, 2012.

94. Li HM, Li J, Wang GZ, et al. Risk factors of 536 cases of gastric cancer[J]. Ningxia Med J, 2013, 35(10): 944-946.

95. Li HX. The Role of H. pylori, Inflammation Cytokine and Related MicroRNA Target SNP in Gastric Cancer[D]. Zhengzhou University, 2011.

96. Li HT. The Study On The Association Of The Ecological Environment And Plasma Trace Elements And Their Related Metabolic Genes SNPs with Gastric Cancer In XianYou County[D]. Fujian Medical University, 2016.

97. Li HZ. A Genome-Wide Association Study of Non-Cardia Gastric Cancer in a Chinese Population[D]. Nanjing Medical University, 2012.

98. Li J. Dietary factors and the relationship between helicobacter pylori and gastric cancer[D].China Medical University, 2009.

99. Li JQ. Ku80 gene G-1401T promoter polymorphism and risk of gastric cancer[D]. Nanjing Medical University, 2011.

100. Li K, Yang J, Cheng ZR. Relationships among Interleukin-1β, Interleukin-1 Receptor Antagonist Gene Polymorphism and Susceptibility to Gastric Cancer[J]. Journal of Sichuan University (Medical Science Edition), 2010(06):122-126.

101. Li K, Dan Z, Liu XB, et al. Epidemiological study on risk factors of gastric cancer among Tibetan population in plateau area[J]. Chin J Public Health, 2013, 29(11): 1613-1615.

102. Li PF. Screening of environmental and genetic risk factors and risk assessment of multiple factors for primary gastric cancer[D]. Southeast University, 2012.

103. Li SQ, Wen Y, Xue QP, et al. TGF-β1 polymorphisms and risk of gastric cancer in a population in Southwestern China[J]. Modern Preventive Medicine, 2015(14):31-34.

104. Li S. Preparation of functional SNP high-throughput detection chip and its application in the study of genetic susceptibility to gastric cancer[D]. Southeast University, 2004.

105. Li SP, Ding JH, Gao CM, et al. A case-control study of esophageal and stomach cancers in high incidence area of upper-digestive tract cancer. [J]. Cancer, 2001(04):46-49.

106. Li T. Relationship between CYP2E1 genetic polymorphism and susceptibility of gastric cancer in Hakkaness[J]. Journal of International Oncology, 2016, 43(7):495-498.

107. Li D, Yang LP, Wang Y, et al. Case control study on risk factors of gastric cancer in xi 'an area[J]. Shaanxi Medical Journal, 2014(3):358-361.

108. Li XC, Xiong JG, Cheng ZW, et al. Association between XPG polymorphisms and risk of gastric cancer[J]. Chinese Journal of Gastroenterology and Hepatology, 2014, 23(3):259-262.

109. Li XC, Xiong JG, Cheng ZW, et al. Association between polymorphism in interleukin-6 and risk of gastric cancer[J]. Chinese Journal of Gastroenterology and Hepatology, 2014, 23(6): 708-711.

110. Li Y, Lu HL, Cao DH, et al. Research on Risk Factors of Gastric Cancer in the Young[J]. Guide of China Medicine, 2016(14):15.

111. Li YF. Study on the effect of mir-200c target polymorphism of EFNA1 gene on the risk of gastric cancer[D]. Guangzhou Medical University, 2013.

112. Li YQ. Interaction of Helicobacter pylori with MDM2 SNP309 polymorphisms and susceptibility to gastric cancer studies[D]. Nanjing Medical University, 2010.

113. Li YT, Guo X, Li YH, et al. Relationship between interleukin - 10, interleukin - 4 Gene polymorphisms and susceptibility of gastric cancer[J]. Clinical Focus, 2015(5):521-524.

114. Li ZT, Xu LD, Liu XD. Study on the relationship between GSH M1 gene polymorphism and genetic susceptibility to gastric cancer[J]. Chinese Journal of Misdiagnostics, 2008(06):58-59.

115. Li ZH. Association between DNA repair gene polymorphisms and environmental factors and Hp-

associated gastric cancer and duodenal ulcer[D]. Nanchang University, 2011.

116. Lian YW. Helicobacter Pylori Infection of Gastric Cancer Group in Xianyou County, The Association of NF-kappa B Non-canonical Signaling Pathway Related Genes SNPs With The Gastric Cancer[D]. Fujian Medical University, 2017.

117. Liang P. Genetic Variation in the PLCE1 Gene Affecting Gene Expression and Relation with the Risk of Gastric Cancer in Chinese Northwestern Han Population[D].Fourth military Medical University, 2014.

118. Lin L. A Case - control Study on Risk Factor of Gastric Cancer and Serological Parameters[D], Fujian Medical University, 2016.

119. Lin XD. Association of Thrombospondins and its SNP with Occurrence and Development of Gastric Carcinoma[D]. Fujian Medical University, 2009.

120. Lin XD, Chen G, Li C, et al. The polymorphisms of matrix metalloproteinase-2 and matrix metalloproteinase-2 tissue inhibitor genes were correlated with genetic susceptibility to gastric cancer[J]. Chinese Journal of Preventive Medicine, 2011(8):711-716.

121. Lin XD, Li C, Shi Y, et al. Nme1-1465 T>C and transformed growth factor beta 1-509t >C gene polymorphism and genetic susceptibility to gastric cancer[J]. Chinese Journal of Pathology, 2010, 39(10):681-685.

122. Lin Y. Study on the relationship between mirna-181a target single nucleotide polymorphism and gastric cancer susceptibility[D]. Guangzhou Medical University, 2013.

123. Ling W. The study on Leptin receptor gene polymorphisms and environmental factors interaction associated with the susceptibility of gastric cancer[D]. Wuhan University, 2016.

124. Liu AM. Epidemiological study on the influencing factors of gastric cancer in high and low incidence areas of malignant tumors in Jiangsu province[D]. Nanjing Medical University, 2007

125. Liu AW, Li K, Yang MY, et al. Correlation of polymorphism of DNA repair gene-XRCC1 with susceptibility to gastric cancer in Shandong Province[J]. Precision Radiation Oncology, 2009, 16(7):489-493.

126. Liu DS. Study on the genetic susceptibility of DNMT3A/3B gene promoter SNPs to gastric cancer and esophageal cancer[D]. Southeast University, 2009.

127. Liu J, XPGHis1104Asp gene polymorphism and risk of gastric cancer[J]. Modern Preventive Medicine, 2014(16).

128. Liu L. Functional FEN1 Genetic Variants Contribute to Risk of Gastrointestinal Cancers[J]. Beijing University of Chemical Technology, 2013.

129. Kiu XM. The study of the relationship between RUNX3 gene polymorphism and the occurrence of gastric cancer[D]. Jiangsu University, 2009.

130. Liu XY. The role of Dsg2 expression and gene single nucleotide polymorphism in the gastric cancer[D]. Fujian Medical University, 2012.

131. Lu F, Qiao L, Han HZ. A case-control study on the relationship between smoking and gastric cancer[J]. Academic Journal of Kaifeng medical college, 1999(03):17-18.

132. Lu B, Xiao XQ, Hong Y, et al. The relationship between h MSH2IVS12-6T ＞ C polymorphism and the risk of gastric cancer [J]. Chinese Journal of Oncology and Surgery, 2010, 02(6):336-339.

133. Lu Y. Genetic polymorphism of immune inflammation and genetic susceptibility to gastric cancer[D]. Nanjing Medical University, 2010.

134. Lu Y, Xu YC, Shen J, et al. Study on the association between the role of polymorphisms of the O6-methylguanine-DNA methyltrans-ferase gene and gastric cancer hereditary susceptibility [J]. Chinese Journal of Disease Control & Prevention, 2006, 10(3):222-225.

135. Lu Y, Xu YC, Shen J, et al. Study on the association between the role of polymorphisms of the O6-methylguanine-DNA methyltrans-ferase gene and gastric cancer hereditary susceptibility [J]. Chinese Journal of Disease Control & Prevention, 2006, 10(3):222-225.

136. Lv HB. To explore the association between TNF-a 857 polymorphism and the risk of gastric cancer[J]. Chinese Journal of Medicinal Guide, 2015(2):180-181.

137. Ma B, Zhang TY, Wan Y, et al. A Case — Control Study of Interleukin — 10 Gene — 592A / C polymorphism and the Risk of Gastric Cancer [J]. Ningxia Medical Journal, 2016, 38(7):580-582.

138. Mai A. Abnormal expression of mir-146a and its single nucleotide polymorphism site rs2910164 were correlated with the susceptibility of gastric cancer[D]. Guangzhou Medical University, 2014.

139.Meng QL, Huang S, Ji GZ, et al. The associations between the Klotho gene G-395A and C1818T polymorphisms and the risk of gastric cancer[J]. Journal of Nanjing Medical University (Natural Sciences), 2008(10):1258-1262.

140. Mu LN, Ding BG, Chen CW, et al. A case-control study on the relationship between methyl-tetra-hydrofolic reductase 677 gene polymorphism and the risk of stomach cancer[J]. Chinese Journal of Epidemiology, 2004, 25(6):495-498.

141. Mu LN, Zhou XF, Ding BG, et al. Genetic polymorphisms of p53 codon72 and the risk of gastric cancer— case-control study [J]. China Oncology, 2003, 13(1):1-4.

142. Mu LN, Zhou XF, Ding BG, et al. A case-control study on drinking green tea and decreasing risk of cancers in the alimentary canal among cigarette smokers and alcohol drinkers[J]. Chinese Journal of Epidemiology, 2003, 24(3):192-195.

143. Ni Q. Epidemiological study of lifestyle factors, caspase-3 and caspase-9 polymorphisms related to gastric cancer[D]. Zhejiang University, 2011.

144. Ni YC. Study on the Association between family history of tumor and gastric cancer risk[D]. Shandong University, 2011.

145. Pan H. Studies on Association of the Overweight/Obesity, Helicobacter Pylori Infection and Gastric Cancer.[D]. Lanzhou University, 2014.

146. Peng CB, Xu R, Xiao SM, et al. Relationship of IFRD1 -rs3807213 Gene Polymorphism with the Risk of Gastric Cancer among Chinese[J]. Journal of Sichuan University (Engineering Science Edition), 2014, 45(1): 49-52.

147. Qian Y. A Molecular Epidemiological Study on Relationship Between the Gastric Polymorphism of Xenobiotic-metabolizing Enzymes and Susceptibility to Gastric Cancer [D].Nanjing Medical University, 2001.

148. Qiu WT. Study on the correlation between animal fat intake and 8-ohdg and the risk of gastric cancer[D]. 2013.

149. Qiu TL, Chen K, Wang XB, et al. A case-control study on the relationship between nutrition and gastric cancer in islanders[J]. Chinese Journal of Epidemiology, 2004, 25(6):497-491.

150. Rao J. Association of Polymorphisms in ERCC and XPF Genes with the Susceptibility to Gastric Cancer[D]. Hebei United University, 2014.

151. Ren XF, Wang J, Yan XL, et al. Relationship between VD R genetic polymorphisms， environmental risk factors and gastric cancer susceptibility: a case-control study [J]. Chinese Journal of Public Health 30(07): 948-951.

152. Shen GS, Zheng FC, Zhao JD. Polymorphisms of PLCE1 rs3765524 among Hui Patients with Gastric Cancer in Qinghai Area[J]. Journal of Qinghai Medical College, 2016, 37(1):36-39.

153. Shen J, Wang RT, Wang LW, et al. The Interaction of H.Pylori CagA Infection and GSTM1 Null Genotype in Stomach Carcinoma [J]. China Cancer, 2003, 12(5):272-276.

154. Shen J, Wang RT, Wang LW, et al. Study on the Relationship between susceptibility of stomach neoplasm cancer and polymorphism of inducible Nitric Oxide Synthase gene[J]. Chinese Journal of Epidemiology, 2002(5).

155. Shen J, Xing HX. Case-control study of the polymorphisms of phase Ⅰ and phase Ⅱ metabolic genes and stomach cancer susceptibility [J]. Tumor, 2002, 22(1):9-13.

156. Shen XB. Environmental and genetic risk factors analysis and risk assessment of primary gastric cancer[D]. Southeast University, 2008.

157. Shi JQ, Luo BJ, Liu RY, et al. Relationship of GSTT1 genotypes and smoking on the susceptibility to gastric cancer[J]. China Medical Herald, 2014, 11(2):63-66.

158. Si PR, Fang DC, Zhang H, et al. Study on the relationship between methylene tetrahydrofolate reductase gene polymorphism and gastric cancer[J]. Chongqing Medical, 2003, 32(9):1123-1125.

159. Song CH, Huang CM, Liu X, et al. Association of-160(C→A)polymorphism in CDH1 gene with gastric cancer risk in Fujian Chinese population[J]. Chinese Journal of Medical Genetics, 2005, 22(5):557-559.

160.Song QQ. Association of C3 and DAF Genetic Variant with the Risk of Gastric Cancer[D]. North China University of Science and Technology, 2016.

161. Sun B. Study on the relationship between igf-1 gene polymorphism and risk of stomach cancer[D]. Zhejiang University, 2007.

162. Sun DL, Duan YN, Zhang XJ, et al. Association between cardiac carcinoma and matrix metalloproteinase-2 and its tissue inhibitor -2 gene polymorphism in the high incidence area of esophageal cancer[J]. Chinese Journal of Preventive Medicine, 2009, 43(4):342-344.

163. Sun JZ, Hu NY, Ying JP, et al. Relationship of EGFR gene rs763317 single nucleotide polymorphism with genetic susceptibility of gastric cancer[J]. Precision Radiation Oncology, 2010, 17(10):724-727.

164.Song P. Association Study of Genetic Variation in the 3’-Untranslated Region of NBN Gene with Gastric Cancer[D]. Soochow University, 2016.

165. Sun T. A six-nucleotide insertion-deletion polymorphism in the CASP8 promoter is associated with susceptibility to multiple cancer[D]. Peking Union Medical College, 2007.

166. Sun XB, Lu JB, Dai DX, et al. A Case-control Study of Relationship of Psychic Factors and Family History of Cancer with Stomach Cancer[J]. Chinese Journal of Prevention and Control of Chronic Diseases, 2001, 9(1):12-14.

167. Sun XW, Dai XD, Lin YJ, et al. he Relationship between Gastric Cancer and the Unhealthy Habits and the Gastric Diseases[J]. Chinese Journal of Prevention and Control of Chronic Diseases, 1999, 7(5):220-222.

168. Xu HR. Epidemiological study on risk factors of gastric cancer[D]. Nanjing Medical University, 2002.

169. Tan Q. Association of Helicobacter Pylori Cellular Receptor DAF Polymorphisms with Gastric Cancer Susceptibility in Chinese Population[D]. Lanzhou University, 2014.

170. Tang XF, He ZD, Li YM, et al. Correlation Between the Nucleotide Polymorphisms of PARP — 1 and the Susceptibility of Gastric Cancer in Hui Ethnic Group of Linxia Hui Autonomous Prefecture[J]. Journal of Medical Research, 2012(03):157-160.

171. Tao M, Zhang LX, Song Y, et al. Association of COX-2 genetic polymorphisms and H． pylori infection with susceptibility of gastric cancer in Shaanxi area[J]. Journal of Shanxi Medical University, 2015(1):17-21.

172. Wang C, Jia ZF, Cao DH, et al. Association of COX-2 genetic polymorphisms and H． pylori infection with susceptibility of gastric cancer in Shaanxi area[J]. Journal of Jilin University (Medicine Edition), 2015, 41(02): 369-373.

173. Wang H. tudy of the relationship between estrogen receptor gene polymorphism and gastric cancer susceptibility of Hui nationality in Qinghai[D]. Qinghai University, 2015.

174. Wang J, Li PF, Fu G, et al. Environment risk factors of stomach cancer among Han people in Nan-Jing: a case-control study[J]. Chin J Public Health. 2012, 28(09): 1137-1139.

175. Wang JG. Analysis of related factors in the development of chronic gastritis to gastric cancer[J]. Hainan Med, 2015(18):47-49.

176. Wang KL, Chen F, Wang XL. Association between Gene Polymorphism in Interleukin-6 and Risk of Gastric Cancer[J]. Modern Preventive Medicine, 2014, 41(18):3450-3452.

177. Wang LN, Ke Q, Chen WS, et al. Polymorphisms of Methylenetetrahydrofolate dehydrogenase gene associated with risk of gastric cancer in south China: a case-control study[C].Cross-strait cancer conference. 2006.

178. Wang LN. The Study on the relationships between the interaction of folate metabolic enzyme environment factors on genetic susceptibility of gastric cancer in Chinese population[D]. Nanjing Medical University, 2007.

179. Wang MH, Chang J, Yu DK, et al. A study on the correlation between ADD1 phosphorylation site missense mutation and the susceptibility of non-cardiac gastric cancer[J]. Chinese Journal of Oncology, 2013, 35(4):311-314.

180. Wang RX, Jia K. Correlation between gene polymorphisms of IL-10-819 and non-cardiac gastric cancer[J]. Chinese Journal of Medicine, 2017(10).

181. Wang XY. Application of the serological test for detection of current Helicobacter pylori infection in Clinic[D]. Nanjing Medical University, 2008.

182. Wang Y, Wen Y, Xue QP, et al. The correlation between PSCA gene polymorphism and risk of gastric cancer[J]. Modern Preventive Medicine, 2016, 43(14):2515-2519.

183. Wang Y, Jiang YH, Sun WJ. Effects of genetic polymorphism of cytochrome P4502 E1 on susceptibility to gastric cancer[J]. Chinese Journal of Public Health, 2005, 21(6):664-665.

184. Wang Z, Gao CM, Wu JZ, et al. The relationship between JWA T723G polymorphism and the risk of gastric cancer[J]. Chinese journal of surgical oncology, 2012, 04(3):148-150.

185. Wei XQ. Correlation between single nucleotide polymorphism of IL-1F5 microRNA target site and the genetic susceptibility of gastric cancer in Henan Han population[D]. Zhengzhou University, 2013.

186. Wei XH, Lu H, Ni JF, et al. Conditional logistic analysis of smoking, alcohol consumption and gastric cancer[J]. Chin J Dis Control Prev, 2006, 10(2):116-119.

187. Wei YH, Zhu GF, Xiao N, et al. A case-control study on the relationship between self-made foods and gastric cancer in rural areas[J]. Chinese Journal of Disease Control and Prevention, 2005, 9(5):387-389.

188. Wen DW, Wang LN, He YT, et al. A Population Based Case -control Study on Risk Factors of Non -cardia Gastric Cancer in Five High –risk Areas in China[J]. China Cancer, 2011(12):11-15.

189. Wu CT, Liu DN, Ma LG, et al. Epidemiological study on risk factors of gastric cancer in coal miners[J]. Modern Preventive Medicine, 2010(08):29-31.

190. Wu D. Association of helicobacter pylori and polymorphisms PSCA, PLCE1 and PRKAA1 with gastric cancer susceptibility[D]. Lanzhou University, 2014.

191. Wu DM. The study between RUNX3 polymorphisms and gastric cancer susceptibility and survival [D]. Nanjing Medical University, 2011.

192. Wu HJ. Correlation of CHRN、CXCL12 TERT-CLPTM1L polymorphism to genetic susceptibility and lymph node metastasis of gastric cancer [D]. Shandong University, 2016.

193. Wu J. Extracellular antagonist of WNT signaling genetic variation and risk of gastric cancer and potential function study [D]. Nanjing Medical University, 2017.

194. Wu J. Molecular Epidemiological study on interleukin2 (IL2), interleukin4 (IL4) and interleukin12B (IL12) and Risk of Gastric Cancer in Chinese Population [D]. Nanjing Medical University, 2009.

195. Wu XQ. Study on the relationship between IL17A and 17F gene polymorphism and gastric cancer [D]. Sun Yat-sen University, 2010.

196. Xiao H. The Association of the TNF, Interleukin-10 gene Polymorphisms with gastric cancer[D]. Wuhan University, 2009.

197. Xiao JR, Chen ZC, Zhou Y, et al. Contrast study on risk factors in the cases of stomach carcinoma [J]. Chinese Journal of Cancer Prevention and Treatment:1854-1856.

198. Xie J, Zhe L, Liu J. Associations of IL-6 / IL-6Ｒ polymorphisms with the risk of gastric cancer in Xuyi region of Huai'an [J]. Chinese Journal of Clinical Laboratory Science, 2017(03):28-32.

199. Xie SQ. Influence of Glutathione S-Transferase M1,T1 Null Genotype and Smoking and Alcohol Consumption on Susceptibility to Gastric Cancer in Zheng Nationality of Guangxi Province [D]. Guangxi Medical University, 2008.

200. Xing PX. Study on the correlation between NF-αand IL-6 gene polymorphism and the susceptibility of gastric adenocarcinoma [D]. Shandong Academy of Medical Sciences, 2005.

201. Xiong F. Association study between genetic polymorphism in CNOT6 and risk of gastric cancer [J].Clinical Focus, 2016. 31(7): 741-744.

202. Xu BF. Association of Genetic Variants on hTERT With Gastric Cancer in a Chinese Population[D]. Qingdao University, 2013.

203. Xu HR. Epidemiological study on risk factors of gastric cancer[D]. Nanjing Medical University, 2002.

204. Xu JC. The study of the correlation of polymorphism of the DNA repair gene- XRCC1 and susceptibility to gastric cancer in Shandong province[D]. Shandong University of Traditional Chinese Medicine, 2006.

205. Xu Q. Study on pri-let-7a polymorphism and susceptibility to gastric cancer and precancerous diseases [D]. 2013.

206. Xu YJ. Association between miR-SNPs of IL23/Th17Pathway Genes and Susceptibility of Gastric Cancer and following Functional Analysis[D]. Zhengzhou University, 2016.

207. Xu, Zhu L, Li SY, et al. To study of Helicobacterium Pylori （HP) Infection and stomach between Xinjiang uygur national minority and Han nationality [J]. Chinese Journal of Laboratory Diagnosis, 2012, 16(5):804-806.

208. Xu LX, Zeng ZR, Wu XQ, et al. Association of the matrix metalloproteinase-9 gene polymorphism with gastric cancer in Guangdong province [J]. Chinese Journal of Gastroenterology:713-716.

209. Xue QP, Pan XF, Li SF, et al. Association between lifestyle factors and behaviors and risk of gastric cancer, Sichuan Province [J]. Modern Preventive Medicine, 2015(07):111-114.

210. Yan YY. Methylation status of socs-1 and RIZ1 genes in primary gastric cancer and analysis of genetic risk factors [D]. 2009.

211. Yan S, Bai ZZ, Zhao JX, et al. CYP2E1RsaⅠgene polymorphism and gastric cancer susceptibility in the study of Qinghai [J]. Journal of Qinghai Medical College, 2013, 34(1):7-14.

212. Yan S, Bai ZZ, Zhao JX, et al. Correlation between genetic polymorphisms of CYP2E1 DraⅠand susceptibility of gastric cancer in Qinghai province [J]. China Oncology, 2013, 23(4):273-278.

213. Yang FH. The epidemiological study on the environmental risk factors of gastric cancer in Xianyou county[D].Fujian Medical University, 2014.

214. Yang F. Risk factors of Gastric cancer and the relationship between risk-factors and the TNF-α[D].Dalian Medical University, 2012.

215. Yang GL. Mechanisms of 1L11 on promoting gastric cancer migration and invasion and the association of IL11 with gastric cancer susceptibility [D]. 2015.

216. Yang J. A Molecular Epidemiological Study on the Relationship between the Genetic Polymorphisms of Interleukin-1 and Susceptibility to Gastric Cancer [D].Nanjing Medical University, 2004.

217. Yang L. Epidemiological study on risk factor of gastric cancer among Buyi population in Qiannan[J]. Public Medical Forum Magazine, 2016, v.20; 460(16): 13-15.

218. Yang J. Functional Analysis and Association Study of SNPs within LncRNA-TUSC7 SNPs and Gastric Cancer Susceptibility[D]. Zhengzhou University, 2018.

219. Yang QY, Jie ZG, Wang J, et al. The correlation between mirna-196a-2 polymorphic site rs11614913 and gastric cancer susceptibility[J]. Academic Journal of Guangzhou Medical University, 2013, 41(3):13-17.

220. Yang T. A 1:1 Case-Control Study on Risk Factors of Gastric cancer [D]. Dalian Medical University, 2008.

221. Yang YF. Study on the interaction of environmental and genetic risk factors in gastric cancer [D]. Southeast University, 2006.

222. Yang J, Huang S, Ji GZ, et al. Association of three sites of single nucleotide polymorphism in Klotho gene with the genetic predisposition of gastric cancer [J]. Journal of Medical Postgraduates, 2013, 26(6):585-588.

223. Ye M, Liu JY, Zhou X, et al. Association between CYE2E1 genotype and genetic susceptibility of gastric cancer [J]. NEGATIVE, 2002, 23(24):2265-2267.

224. Yin ZZ, Wang XY, Jiang XJ, et al. Relationship between IL-17 gene polymorphism and gastric cancer[J]. Chinese Journal of Oncology Prevention and Treatment , 2017,9(01): 50-54.

225. Yi D. Study on relationship between inflammatory cytokine gene polymorphisms and susceptibility of IL-1B 、IL-1RN、TNF-A gastric cancer, and prognosis in uygur[D].Xinjiang Medical University, 2011.

226. Yu H. Role of ERCC1 and ERCC2 genetic polymorphisms in the gastric cancer susceptibility and prognosis of gastric cancer patients receiving oxaliplatin-based chemotherapy [D]. Southern Medical University, 2017.

227. Yuan Q, Xue YD, Zheng YP, et al. Investigation on ALDH2 gene polymorphism and gastric cancer susceptibility of Han nationality in Jinhua, Zhejiang [J]. Chinese Journal of Infection Control:153-155.

228. Yue ZY. Association of heparanase gene (HPSE-1) single nucleotide polymorphisms with gastric cancer[D]. China Medical University, 2010.

229. Zang J. Macrophage migration in history factor and tumor necrosis factor-alpha promoter polymorphisms in gastric cancer susceptibility in case and control family members [D]. Zhengzhou University, 2009.

230. Zang JY, Liu WT, Zang JY, et al. Risk Factors for Gastric Cancer in Tianjin Area: A Case -control Study [J]. Chinese Journal of Prevention and Control of Chronic Non-Communicable Diseases, 2011, 19(2):138-140.

231. Zhang CZ. Correlation between XPD gene polymorphism and gastric cancer susceptibility[D]. Shandong University, 2010.

232. Zhang D. Study on the correlation between cox-2 and parp-1 gene polymorphism and gastric cancer [D]. Lanzhou University, 2009.

233. Zhang HP and Zhang H. The relationship between the polymorphism of il-1rn and tnf-a-308 gene and the susceptibility to gastric cancer[J]. ACTA Universityis Medicinalis Anhui, 2011(04):45.

234. Zhang HL. Study on the correlation between the promoter region and coding region SNPs of il-1 beta gene and gastric cancer susceptibility [D]. 2007.

235. Zhang J. Study on genetic polymorphism of metabolic enzymes and environmental exposure associated with gastric cancer [D]. Southeast University, 2004.

236. Zhang JH. Relationship between Polymorphisms of Interleukin-8 and Susceptibility of Gastric Cancer in Henan Han Population[D]. Zhengzhou University, 2012.

237. Zhang JX. The study on the relationship between genetic polymorphisms of IL-1,TNF and helicobacter pylori-induced gastric cancer in a Henan population[D]. Zhengzhou University, 2007.

238. Zhang LY. Association study of MMP-2 and TIMP-2 and its single nucleotide polymorphisms with gastric carcinoma[D]. Fujian Medical University, 2011.

239. Zhang MJ. Correlation of FAS and FASL gene Polymorphisms with susceptibility to esophageal carcinoma[D]. Shandong University, 2011.

240. Zhang Q. Association studies of polymorphisms in microRNA and it’s biological processessing genes with the susceptibility of gastric cancer [D]. Nanjing Medical University, 2012.

241. Zhang QB. Association between PARP-1 polymorphisms and susceptibility to gastric cancer[D]. Lanzhou University, 2007.

242. Zhang TT. Study on risk factors of gastric cancer and related markers of helicobacter pylori [D]. Harbin Medical University, 2012.

243. Zhang WQ, Zhao J, Liu JF, et al. Risk of cardiac and gastric cancer and gene polymorphisms of matrix metalloproteinase (MMP)-9 in northwestern Chinese population of Han nationality of China [J]. China Journal of Modern Medicine, 2008(22):66-69.

244. Zhang WT, Liang P, Dai P, et al. Evaluation of gene polymorphisms associated with gastric cancer susceptibility by Logistic regression and multifactor dimensionality reduction analysis [J]. Chinese Journal of Biologicals, 27(05):679-683.

245. Zhang X. Study on the relationship between dinner-to-bed time, post dinner walk and gastric cancer [D]. Fujian Medical University, 2016.

246. Zhang XM. Functional genetic variation in cyclooxygenase-2 and their association with susceptibility to gastroesophageal cancer[D]. Peking Union Medical College, 2006.

247. Zhang YM, Ma JH, Liu YC, et al. Correlation Study on miR-449a Gene Polymorphism of Single Nucleotide and Susceptibility to Gastric Cancer in A Chinese Han Population [J]. Journal of Modern Laboratory Medicine, 2016, 31(4):14-18.

248. Zhang YL. A Case-Control Study on Risk Factors of Gastric cancer in Yancheng [D]. Southeast University, 2016.

249. Zhang Y. The relationship of polymorphism of the DNA repair gene- XRCC1 and susceptibility to gastric cancer in Shandong province[D]. Shandong University of Traditional Chinese Medicine, 2007.

250. Zhang HZ. Association study of genetic variants at chromosome 1q22, 10q23, 20p13 and 8q24 with susceptibility of gastric cancer[D]. Nanjing Medical University, 2011.

251. Zhao JX. Preliminary Research on Co-relationship between Genetic Polymorphisms of CYP2E1 and

Susceptibility of Gastric Cancer in Qinghai Province[D]. Qinghai University, 2012.

252. Zhao J, Lin Y, Nie YQ, et al. Polymorphism of 1670C>T in MTMR3 is Associated with the Susceptibility to Gastric Cancer [J]. Progress in Modern Biomedicine, 2016(2):221-224.

253. Zhao XM. Genetic polymorphism of IRS-1， IRS-2 and susceptibility to gastric cancer[D]. Nanjing Medical University, 2012.

254. Zhao Y, Chen F, Yang SJ, et al. Association of single nucleotide polymorphisms of CMA and ALDH2 with the risk of gastric cancer [J]. Modern Preventive Medicine, 2014, 41(21):3975-3978.

255. Zheng LZ. Study on the relationship between interleukin-1 gene (il-1) polymorphism and genetic susceptibility to gastric cancer [D].2007.

256.Zheng TR, Zheng QH, Long FS, et al. GSTT1 and GSTM1 gene deletion polymorphism and gastric cancer susceptibility [J]. Journal of Practical Oncology, 2002(03):14-16.

257. Zheng ZL. CYPZEI, GSTMI, GSTTI Polymorphisms and Susceptibilities to Cardia and Non-cardia Gastric Cancer in High-Risk Area of China[D]. Fujian Medical University, 2003.

258. Zhou JN, Gao CM, TakezakiToshiro, et al. Interaction between polymorphisms in CYP2E1 RSA Ⅰ genotypes and lifestyle with risk of stomach cancer [J]. Journal of Oncology, 2003, 9(5):285-288.

259. Zhou T, Fan W, Han W, et al. Genetic polymorphism, common risk factors and susceptibility to gastric cancer [J]. Chinese Journal of Digestion, 2007, 27(3).

260. Zhou Y, Jin GF, Jiang GJ, et al. Correlations of Polymorphisms of TGFB1 and TGFBR2 Genes to Genetic Susceptibility to Gastric Cancer [J]. Chinese Journal of Cancer, 2007, 26(6):581-585.

261. Zhou Y, Wang LN, Jiang GJ, et al. Molecular epidemiological study on the relationship between polymorphism of reduced folate carrier gene RFC1-G 80A and susceptibility of gastric cancer[J]. Tumor, 2006, 26(12):1081-1084.

262. Zhou Y. Helicobacter Pylori Infection in Xianyou County、the Association of Nucleotide Polymorphism of Inflammation Factors related to miRNA Targe Site with The Gastric Cancer [D]. Fujian Medical University, 2016.

263. Zhou Y. Association of ADIPOQ gene polymorphisms and environmental factor with risk of gastric cancer in Chinese population from north-west China [D]. Lanzhou University, 2011.

264. Zhou Y. Association analysis of genetic variants EZH2 and MiRNA network gene with risk gastric cancer risk [D]. Anhui Medical University, 2012.

265. Zhou YF, Wu MH, Xie YR. Et al. A case control study of gastric cancer risk factors in Lishui City [J]. China Medical Herald, 2018(24):15(24): 45-50.

266. Zhou ZY, Li JQ, Sun GX, et al. Genetic polymorphisms of ADH2,ALDH2 and alcohol drinking investigated for their connection with stomach cancer[J]. Shanghai Journal of Preventive Medicine, 2010, 22(4):207-209.

267. Zhu HX. Study on the Association of XRCC1 and SMAD4 Polymorphisms and the Susceptibility of Gastric Cancer [D]. Nanjing Medical University, 2009.

268. Zhu HJ. A study on the association between MPOG-463A polymorphism and susceptibility to gastrointestinal cancer[D]. Nanjing Medical University, 2007.

269. Zhu KX. Study on the relationship among genetic polymorphism of COX-2 and P53, H. pylori infection and susceptibility to gastric cancer in high incidence area [D]. Lanzhou University, 2008.

270. Zhuang SL, Liu Y, Zhu HM, et al. A case-control study of gastric cancer risk factors in Nanjing Pukou district [J]. Jiangsu Journal of Preventive Medicine, 2013, 24(5):6-8.

271. Chen B, Luo HL, Lu J, et al. Genetic Epidemiology Study on Upper Gastrointestinal Carcinoma in Wuwei City of Gansu Province[J]. China Cancer, 2019, 28(4):264-270.

272. Miao CF, Ma YT, Wang XP. J, et al. Construction of the risk factor model of gastric cancer and its value in screening opportunity and options[J]. CHINA ONCOLOGY, 2019, 29(7):501-507.

273. Yin XL. Changes in BMI and body shap in relation to risk of gastric cancer: A population-based case-control study in China[D]. Shandong University, 2019.

274. Mei Y. Case-control study on risk factors of esophageal cancer and gastric cancer in Danjiangkou City[D]. Wuhan University of Science and Technology, 2019.

275. Zhu Q. Risk factors gastric cancer of rural residents central and eastern parts of gansu province: A case-control study[D]. Lanzhou University, 2019.

276. Hao RT, Dong SX, Wang L, et al. A Case-control Study on Ｒisk Factors of Gastric Cancer in Sheyang County of Jiangsu Province.2019, 9(1):49:55.

277. Gao LL,Song G. Relationship between CCNDI Gene Polymorphism and Gastric Cancer[J] 2019,48(1):126-130.

278. Liu JY, Yan HF, Zhang YJ. Genetic susceptibility of gastric cancer with single nucleotide polymorphisms(SNPs)of apoptotic genes - caspase - 3 and caspase -7[J]. 2019(3):250-255.

279. Gao F,Ma LC, Dong WJ, et al. Analysis on association between single nucleotide polymorphisms of ITGA1 gene and risk of non-cardia gastric cancer[J]. Journal of Jilin University (Medicine Edition).2019,45(3):577:581.

280. Tan RX. Relationship between NLRP3 gene polymorphism and genetic susceptibility to chronioc gastritis and gastric adenocarcinoma[D]. Guangxi Medical University, 2019.

281. Zhang QS, Zhu CW, Wang SL, et al. Susceptibility of polymorphisms in integrins to risk of gastric cancer[J]. The Journal of Practical Medicine,2019, 35(8):1282-1287.

282. Song XX, Wang SS, Guo J, et al. Case-Control Study of Risk Factors for Gastric Cancer[J]. Advances in Clinical Medicine. 2020,10(4):537-545.

283. Guo F , Xu Q , Lv Z , et al. Correlation Between TNFAIP2 Gene Polymorphism and Prediction/Prognosis for Gastric Cancer and Its Effect on TNFAIP2 Protein Expression[J]. Frontiers in Oncology, 2020, 10.

284. Gao F, Ru WX, Ma LC, et al. Association between TLR5 gene polymorphism and non-cardiac gastric cancer[J]. Acta Universitatis Medicinalis Anhui, 2020, 55(1):114-117.

285. Yuan L, Zhang LW, Er LM, et al. Research on genetic susceptibility of Bcl-2 gene polymorphism to gastric cardiac carcinoma and precancerous lesions in population with high incidence of esophageal cancer[J]. Chinese Journal of Cancer Prevention and Treatment, 2019, 26(22): 1669-1674.

286. Shu ZX, Wu CC, Wu XL, et al. Relationships between gastric cancer and SNPs of autophagy-related genes on the PI3K/Akt/mTOR pathway[J]. Crcinogenesis, teratogenesis and mutagenesis, 2020, 32(2):118-125.

287. Ying W, Wang XY, Jiang B, et al. Long non-coding RNA H19 genetic polymorphisms and susceptibility of gastric cancer[J]. ONCOLOGY PROGRESS, 2020, 18(14):1427-1430.

288. Li D, Liu BY, Yu MM, et al. NFKBIA polymorphism, eating habits and susceptibility to gastric cancer: a case-control study[J]. Crcinogenesis, teratogenesis and mutagenesis, 2020, 32(3):198-201.

289. Du J , Xu Y , Dai J , et al. Genetic variants at 5p15 are associated with risk and early onset of gastric cancer in Chinese populations[J]. Carcinogenesis, 2013, 34(11):2539-2542.

290. Du M , Wang W , Jin H , et al. The association analysis of lncRNA HOTAIR genetic variants and gastric cancer risk in a Chinese population[J]. Oncotarget, 2015, 6(31): 31255-31262.

291. Abnet C C , Zheng W , Ye W , et al. Plasma pepsinogens, antibodies against Helicobacter pylori, and risk of gastric cancer in the Shanghai Women\"s Health Study Cohort[J]. Br J Cancer, 2011, 104(9):1511-1516.

292. Abnet C C , Freedman N D , Hu N , et al. A shared susceptibility locus in PLCE1 at 10q23 for gastric adenocarcinoma and esophageal squamous cell carcinoma[J]. Nature Genetics, 2010, 42(9):764-767.

293. Bai, Y, Li H, WangL, et al. Role of ERCC5 His1104Asp and His46His gene polymorphisms in the development of gastric cancer risk in a Chinese Han population. INTERNATIONAL JOURNAL OF CLINICAL AND EXPERIMENTAL PATHOLOGY, 2016, 9(3): 3925-3930.

294. Cai H , Ye F , Michel A , et al. Helicobacter pylori\r, blood biomarker for gastric cancer risk in East Asia[J]. International Journal of Epidemiology, 2016, 45(3): 774-81.

295.Cai, L. and S. Z. Yu, et al. Cytochrome P450 2E1 genetic polymorphism and gastric cancer in Changle, Fujian Province. World J Gastroenterol, 2001, 7(6): 792-5.

296. Cai, L. and Z. Zhang. A case-control study on the risk factor of cardia cancer in Fujian Province. Cancer Research on Prevention and Treatment, 2003, 30(1): 62-64.

297. Cai, M. and Y. Zhang, et al. Association between microRNA-499 polymorphism and gastric cancer risk in Chinese population. Bull Cancer,2015, 102(12): 973-8.

298. Cao, B. and L. Zhu, et al. Genetic variations and haplotypes in TIM-3 gene and the risk of gastric cancer. Cancer Immunology, Immunotherapy, 2010, 59(12): 1851-1857.

299. Cao, H. X. and S. P. Li, et al. Alcohol dehydrogenase-2 and aldehyde dehydrogenase-2 genotypes, alcohol drinking and the risk for stomach cancer in Chinese males. Asian Pac J Cancer Prev, 2010, 11(4): 1073-7.

300. Cao, N. and M. Li, et al. rs61991156 in miR-379 is associated with low capability of glycolysis of gastric cancer by enhanced regulation of PKM2. Cancer Cell International, 2018, 18(1).

301. Chang, S. C. and P. Y. Chang, et al. Single nucleotide polymorphisms of one-carbon metabolism and cancers of the esophagus, stomach, and liver in a Chinese population. PLoS One, 2014, 9(10): e109235.

302. Chang, S. C. and B. Y. Goldstein, et al. Plasma folate, vitamin B12, and homocysteine and cancers of the esophagus, stomach, and liver in a Chinese population. Nutr Cancer, 2015, 67(2): 212-23.

303. Chen, B. and J. Wang, et al. The MDM2 rs937283 A > G variant significantly increases the risk of lung and gastric cancer in Chinese population. International Journal of Clinical Oncology, 2018, 23(5): 867-876.

304. Chen, B. and Z. Zeng, et al. IL23R +2199A/C polymorphism is associated with decreased risk of certain subtypes of gastric cancer in Chinese: a case-control study. Cancer Epidemiol, 2011, 35(2): 165-9.

305. Chen, H. and L. Chen, et al. The Kruppel-like factor 6 genotype is associated with gastric cancer in a Chinese population. J Int Med Res, 2010, 38(5): 1801-7.

306. Chen, J. and Y. Lu, et al. A nonsynonymous polymorphism in IL23R gene is associated with risk of gastric cancer in a Chinese population. Mol Carcinog, 2010, 49(10): 862-8.

307. Chen, J. and L. Miao, et al. TGFBR1 tagging SNPs and gastric cancer susceptibility: a two-stage case-control study in Chinese population. Mol Carcinog, 2014, 53(2): 109-16.

308. Chen, J. and N. Liu, et al. Association between ITGA2 C807T polymorphism and gastric cancer risk. WORLD JOURNAL OF GASTROENTEROLOGY, 2011,17(23): 2860-2866.

309. Chen, L. Z. and C. Y. He, et al. SPP1 rs4754 and its epistatic interactions with SPARC polymorphisms in gastric cancer susceptibility. Gene, 2018, 640: 43-50.

310. Chen, M. and B. Jiang, et al. Genetic variations in PRKAA1 predict the risk and progression of gastric Cancer. BMC Cancer, 2018,18(1): 923.

311. Chen, Q. and R. Qin, et al. A functional variant at the miR-214 binding site in the methylenetetrahydrofolatereductase gene alters susceptibility to gastric cancer in a chinese han population. Cellular Physiology and Biochemistry, 2015, 36(2): 622-630.

312. Chen, S. Y. and T. Y. Liu, et al. Modification effects of GSTM1, GSTT1 and CYP2E1 polymorphisms on associations between raw salted food and incomplete intestinal metaplasia in a high-risk area of stomach cancer. INTERNATIONAL JOURNAL OF CANCER, 2004, 108(4): 606-612.

313. Chen, S. and Z. Zheng, et al. Association of polymorphisms and haplotype in the region of TRIT1, MYCL1 and MFSD2A with the risk and clinicopathological features of gastric cancer in a southeast chinese population. Carcinogenesis, 2013, 34(5): 1018-1024.

314. Chen, S. and X. C. Zhu, et al. Investigating the association between XRCC1 gene polymorphisms and susceptibility to gastric cancer. Genet Mol Res, 2016, 15(3).

315. Chen, W. and L. Wang, et al. The role of IGFBP3 functional polymorphisms in the risk of gastric cancer in a high-risk Chinese population. Eur J Cancer Prev, 2008, 17(2): 82-7.

316. Chen, X. and G. Wang, et al. Polymorphisms and haplotypes of the miR-148/152 family are associated with the risk and clinicopathological features of gastric cancer in a Northern Chinese population. Mutagenesis, 2014, 29(6): 401-7.

317. Chen, X. and Y. Xu, et al. Associations of Il-1 Family-Related Polymorphisms With Gastric Cancer Risk and the Role of Mir-197 In Il-1f5 Expression. Medicine (Baltimore) , 2015,94(47): e1982.

318. Chen, Y. Z. and F. Guo, et al. Association between XPG polymorphisms and stomach cancer susceptibility in a Chinese population. J Cell Mol Med, 2016, 20(5): 903-8.

319. Chen, Z. H. and J. F. Xian, et al. Analysis of ADH1B arg47his, ALDH2 glu487lys, and CYP4502E1 polymorphisms in gastric cancer risk and interaction with environmental factors. Genetics and Molecular Research, 2016, 15(4).

320. Chen, Z. and C. Zhang, et al. Effects of selected genetic polymorphisms in xeroderma pigmentosum complementary group D on gastric cancer. MOLECULAR BIOLOGY REPORTS, 2011, 38(3): 1507-1513.

321. Chiu, C. F. and C. H. Wang, et al. A novel single nucleotide polymorphism in XRCC4 gene is associated with gastric cancer susceptibility in Taiwan. Ann Surg Oncol, 2008,15(2): 514-8.

322. Chu, H. and Q. Zhao, et al. Tagging SNPs in the ERCC4 gene are associated with gastric cancer risk. Gene, 2013,521(1): 50-4.

323. Cong, L. and W. B. Wang, et al. Foki polymorphism of the vitamin d receptor gene is associated with susceptibility to gastric cancer: A case-control study. Tohoku Journal of Experimental Medicine, 2015, 236(3): 219-224.

324. Dedong, H. and Z. Bin, et al. The contribution of the genetic variations of the matrix metalloproteinase-1 gene to the genetic susceptibility of gastric cancer. Genet Test Mol Biomarkers, 2014, 18(10): 675-82.

325. Dong, H. and X. Jin, et al. High gamma-Radiation Sensitivity Is Associated with Increased Gastric Cancer Risk in a Chinese Han Population: A Case-Control Analysis. PLOS ONE, 2012,7(8).

326. Dong, Y. and J. Chen, et al. Evaluating the Association of Eight Polymorphisms with Cancer Susceptibility in a Han Chinese Population. PLoS One, 2015, 10(7): e0132797.

327 Du, J. and X. Zhu, et al. Telomere length, genetic variants and gastric cancer risk in a Chinese population. CARCINOGENESIS, 2015, 36(9): 963-970.

328. Duan, X. and W. Cao, et al. Genetic variants in TERT are associated with risk of gastric cancer in a Chinese Han population. Oncotarget, 2016, 7(50): 82727-82732.

329. Duan, Z. and C. He, et al. Promoter polymorphisms in DNA repair gene ERCC5 and susceptibility to gastric cancer in Chinese. Gene, 2012, 511(2): 274-9.

330. Elingarami, S. and H. Liu, et al. Polymorphisms in NEIL-2, APE-1, CYP2E1 and MDM2 Genes are Independent Predictors of Gastric Cancer Risk in a Northern Jiangsu Population (China). J Nanosci Nanotechnol, 2015, 15(7): 4815-28.

331. Elingarami, S. and Y. Deng, et al. NEIL-2 Single Nucleotide Polymorphism Genotyping Using Single Base Extension on Core-Shell Fe3O4@SiO2@Au Magnetic Nanoparticles and Association of the Genotypes with Gastric Cancer Risk in Northern Jiangsu (China). SCIENCE OF ADVANCED MATERIALS, 2014, 6(5): 899-907.

332. Epplein, M. and W. Zheng, et al. Prospective study of Helicobacter pylori biomarkers for gastric cancer risk among Chinese men. Cancer Epidemiol Biomarkers Prev, 2012, 21(12): 2185-92.

333. Epplein, M. and Y. Xiang, et al. Circulating cytokines and gastric cancer risk. CANCER CAUSES & CONTROL, 2013, 24(12): 2245-2250.

334 Fan, H. and D. Liu, et al. A functional polymorphism in the DNA methyltransferase-3A promoter modifies the susceptibility in gastric cancer but not in esophageal carcinoma. BMC Med, 2010, 8:12.

335. Fan, Q. H. and R. Yu, et al. The has-miR-526b binding-site rs8506G>a polymorphism in the lincRNA-NR_024015 exon identified by GWASs predispose to non-cardia gastric cancer risk. PLoS One, 2014, 9(3): e90008.

336. Feng, Y. B. and D. Q. Fan, et al. Association between XPG gene polymorphisms and development of gastric cancer risk in a Chinese population. Genet Mol Res, 2016, 15(2).

337. Gao, C. and T. Takezaki, et al. Interaction between cytochrome P-450 2E1 polymorphisms and environmental factors with risk of esophageal and stomach cancers in Chinese. Cancer Epidemiol Biomarkers Prev, 2002, 11(1): 29-34.

338. Gao, S. and X. Zhang, et al. Genetic epidemiological analysis reveals a multi-gene additive model for gastric cancer. FAMILIAL CANCER, 2011, 10(1): 119-125.

339. Yugang G , Yu H , Mingkun J , et al. Polymorphisms in lncRNA PTENP1 and the Risk of Gastric Cancer in a Chinese Population[J]. Disease Markers, 2017, 2017:1-8.

340. Yuqiu G , Hanting L , Xiaonan Q , et al. Genetic variants in PI3K/Akt/mTOR pathway genes contribute to gastric cancer risk[J]. Gene, 2018, 670:130-135.

341. Gong H , Li H , Zou J , et al. The relationship between five non-synonymous polymorphisms within threeXRCCgenes and gastric cancer risk in a Han Chinese population[J]. Tumor Biology, 2016, 37(5):5905-5910.

342. Gong, Y. and C. He, et al. (2013). Association of two ERCC4 tagSNPs with susceptibility to atrophic gastritis and gastric cancer in Chinese. Gene 519(2): 335-42.

343. Dongying G , Meilin W , Shizhi W , et al. The DNA Repair Gene APE1 T1349G Polymorphism and Risk of Gastric Cancer in a Chinese Population[J]. PLoS ONE, 2011, 6(12):e28971.

344. Gu H , Ni M , Guo X , et al. The functional polymorphism in monocyte chemoattractant protein-1 gene increases susceptibility to gastric cancer[J]. Medical Oncology, 2011, 28(1 Supplement):280-285.

345. Gu H , Yang L , Sun Q , et al. Gly82Ser polymorphism of the receptor for advanced glycation end products is associated with an increased risk of gastric cancer in a Chinese population.[J]. Clinical Cancer Research, 2008, 14(11):3627-3632.

346. Gu J Y, Tu L. Investigating the role of polymorphisms in miR-146a, -149, and -196a2 in the development of gastric cancer[J]. Genetics & Molecular Research Gmr, 2016, 15(2).

347. Role of polymorphisms of the IGF2 and IGFBP3 genes and risk of gastric carcinoma in China[J]. Chinese Medical Journal 127(3): 412-416.

348. Gu Y , Deng B , Kong J , et al. Functional polymorphisms in NR3C1 are associated with gastric cancer risk in Chinese population[J]. Oncotarget, 2017, 8(62): 105312-105319.

349. Guo, W. and Z. Dong, et al. (2012). Association of polymorphisms in transforming growth factor-beta receptors with susceptibility to gastric cardia adenocarcinoma. MOLECULAR BIOLOGY REPORTS 39(4): 4301-4309.

350. Guo W , Dong Z , Guo Y , et al. Association of polymorphisms in transforming growth factor-β receptors with susceptibility to gastric cardia adenocarcinoma[J]. Molecular Biology Reports, 2012, 39(4):4301-4309.

351. He B S , Pan Y Q , Xu Y F , et al. Polymorphisms in Interleukin-1B (IL-1B) and Interleukin 1 Receptor Antagonist (IL-1RN) Genes Associate with Gastric Cancer Risk in the Chinese Population[J]. Digestive Diseases & Sciences, 2011, 56(7):2017-2023.

352. Bang-Shun H , Hui-Ling S , Tao X , et al. Association of Genetic Polymorphisms in the LncRNAs with Gastric Cancer Risk in a Chinese Population[J]. Journal of Cancer, 2017, 8(4):531-536.

353 He B , Pan Y , Xu Y , et al. Increased risk for gastric cancer in carriers of the lymphotoxin-α+252G variant infected by Helicobacter pylori.[J]. Genetic Testing & Molecular Biomarkers, 2012, 16(1):9.

354. He B , Xu T , Pan B , et al. Polymorphisms of TGFBR1, TLR4 are associated with prognosis of gastric cancer in a Chinese population[J]. Cancer Cell International, 2018, 18(1).

355. He C , Tu H , Sun L , et al. Helicobacter pylori-related host gene polymorphisms associated with susceptibility of gastric carcinogenesis: a two-stage case-control study in Chinese[J]. Carcinogenesis, 2013, 34(7):1450-1457.

356. He, Jing, Qiu, et al. Polymorphisms in the XPG gene and risk of gastric cancer in Chinese populations[J]. Human Genetics, 2012, 131(7):1235-1244.

357. Genetic variations of mTORC1 genes and risk of gastric cancer in an eastern chinese population[J]. Molecular Carcinogenesis, 2013, 52(S1):70-79.

358. He N , Liu L , Duan X , et al. Identification of a shared protective genetic susceptibility locus for colorectal cancer and gastric cancer[J]. Tumor Biology, 2015, 37(2):2443-2448.

359. He W , Liu T , Shan Y , et al. PARP1 Polymorphisms Increase the Risk of Gastric Cancer in a Chinese Population[J]. MOLECULAR DIAGNOSIS & THERAPY, 2012, 16(1):35-42.

360. He X , Qiao Q , Ge N , et al. Irradiation-induced telomerase activity and gastric cancer risk: a case-control analysis in a Chinese Han population[J]. Bmc Cancer, 2010, 10(1):312-320.

361. Hong Y , Ge Z , Jing C , et al. Functional Promoter -308G>A Variant in Tumor Necrosis Factor α Gene Is Associated with Risk and Progression of Gastric Cancer in a Chinese Population[J]. PLoS One 8(1): e50856.

362. Hou C , Yang F . Interleukin-17A gene polymorphism is associated with susceptibility to gastric cancer.[J]. International Journal of Clinical & Experimental Pathology, 2015, 8(6):7378-84.

363. Hu J F , Zhang S F , Jia E M , et al. Diet and cancer of the stomach: A case-control study in China[J]. International Journal of Cancer, 1988, 41(3):331-335. 364. Hua R X , Zhuo Z J , Shen G P , et al. Polymorphisms in the XPC gene and gastric cancer susceptibility in a Southern Chinese population[J]. OncoTargets and Therapy, 2016, Volume 9:5513-5519.

365. Hua T , Qinsheng W , Xuxia W , et al. Nuclear Factor-Kappa B1 is Associated With Gastric Cancer in a Chinese Population[J]. Medicine, 2014, 93(28):e279.

366. Huan X , He Y , Huang C , et al. Functional Genetic Variants in SPHK1 Affect Susceptibility to Gastric Cancer in a Chinese Population[J]. Clinical Laboratory, 2017, 63(9):1347-1356.

367. Yang L , Huang C , Wang Y , et al. Association analysis of DACT1 genetic variants and gastric cancer risk in a Chinese Han population: a case–control study[J]. OncoTargets and Therapy, 2016, Volume 9:5975-5983.

368. A 5'-flanking region polymorphism in toll-like receptor 4 is, associated with gastric cancer in a Chinese population[J]. Journal of Biomedical Research 24(2): 100-106.

369. Huang L , Yuan K , Liu J , et al. Polymorphisms of the TLR4 gene and risk of gastric cancer[J]. Gene, 2014, 537(1):46-50.

370. Huang T , He K , Mao Y , et al. Genetic variants in PPP2CA are associated with gastric cancer risk in a Chinese population[J]. Scientific Reports, 2017, 7(1).

371. Hussain S K , Mu L N , Cai L , et al. Genetic Variation in Immune Regulation and DNA Repair Pathways and Stomach Cancer in China[J]. Cancer Epidemiology Biomarkers & Prevention, 2009, 18(8):2304-2309.

372. Genetic variants in fas signaling pathway genes and risk of gastric cancer[J]. International Journal of Cancer, 2014, 134(4):822-831.

373. Jeon C , Chang S C , Mu L , et al. Genetic Variants of Peroxisome Proliferator-Activated Receptor δ Are Associated with Gastric Cancer[J]. Digestive Diseases and Sciences, 2013, 58(10):2881-2886.

374. Ji H X , Chang W S , Tsai C W , et al. Contribution of DNA Repair Xeroderma Pigmentosum Group D Genotype to Gastric Cancer Risk in Taiwan[J]. Anticancer research, 2015, 35(9):4975-4981.

375. Jia A , Gong J , Li Y , et al. GG genotype of cyclin D1 G870A polymorphism is associated with non-cardiac gastric cancer in a high-risk region of China[J]. Scandinavian Journal of Gastroenterology, 2008, 43(11):1353-1359.

376. Jiang H , Wang H , Ge F , et al. The Functional Variant in the 3 ' UTR of IGF1 with the Risk of Gastric Cancer in a Chinese Population[J]. Cellular Physiology and Biochemistry, 2015, 36(3):884-892.

377. Jin G , Miao R , Deng Y , et al. Variant genotypes and haplotypes of the epidermal growth factor gene promoter are associated with a decreased risk of gastric cancer in a high-risk Chinese population[J]. Cancer science, 2007, 98(6):864-868.

378. Jin G , Wang L , Chen W , et al. Variant alleles ofTGFB1 andTGFBR2 are associated with a decreased risk of gastric cancer in a Chinese population[J]. International Journal of Cancer, 2007, 120(6):1330-1335.

379. Jing C , Huang Z J , Duan Y Q , et al. Glulathione-S-transferases gene polymorphism in prediction of gastric cancer risk by smoking and Helicobacter pylori infection status[J]. Asian Pacific Journal of Cancer Prevention Apjcp, 2012, 13(7):3325-8.

380. Ke Q , Liang J , Wang L N , et al. Potentially functional polymorphisms of the vascular endothelial growth factor gene and risk of gastric cancer[J]. Molecular carcinogenesis, 2008, 47(8):647-651.

381. Lee, W.-P. The -251T Allele of the Interleukin-8 Promoter Is Associated with Increased Risk of Gastric Carcinoma Featuring Diffuse-Type Histopathology in Chinese Population[J]. Clinical Cancer Research, 2005, 11(18):6431-6441.

382. Li B , Liu H Y , Guo S H , et al. Mll3 Genetic Variants Affect Risk of Gastric Cancer in the Chinese Han Population[J].

Asian Pacific journal of cancer prevention: APJCP, 2013, 14(7):4239-4242.

383. Li B , Liu H Y , Guo S H , et al. A missense mutation (S3660L) in MLL3 gene influences risk of gastric cancer.[J]. Journal of Buon, 2014, 19(2):394-397.

384. Li B , Liu H , Gong F , et al. Molecular Epidemiologic Correlation Analysis Between Caspase3 Gene Polymorphism and Gastric Cancer Susceptibility[J]. Cell Biochemistry and Biophysics, 2014, 70(3):1647-1653.

385. Li F , Zhong M Z , Li J H , et al. Case-control study of single nucleotide polymorphisms of PSCA and MUC1 genes with gastric cancer in a Chinese.[J]. Asian Pacific Journal of Cancer Prevention Apjcp, 2012, 13(6):2593.

386. Li H , Ren C , Fan Z , et al. A Genetic Variant in 3′-Untranslated Region of Cyclooxygenases-2 Gene Is Associated with Risk of Gastric Cancer in a Chinese Population[J]. DNA and Cell Biology, 2012, 31(7):1252-1257.

387. Li J, Ma G, Zhu X, et al. Association analysis of telomere length related gene ACYP2 with the gastric cancer risk in the northwest Chinese Han population[J]. Oncotarget, 2017, 8(19):31144-31152.

388. Association of CD14/-260 polymorphism with gastric cancer risk in Highland Tibetans[J]. World J Gastroenterol , 20(10): 2688-94.

389. Li L, Jia F, Bai P, et al. Association between polymorphisms in long non-coding RNA PRNCR1 in 8q24 and risk of gastric cancer. Tumor Biology, 2016, 37(1): 299-303.

390. Li L , Tang XY , Ye LM , et al. Investigation on the association between IL-10 C819T gene polymorphisms and susceptibility to gastric cancer[J]. Genetics and molecular research: GMR, 2016, 15(4).

391. Li M , Li R J , Bai H , et al. Association between the pre-miR-196a2 rs11614913 polymorphism and gastric cancer susceptibility in a Chinese population[J]. Genetics and molecular research: GMR, 2016, 15(2).

392. Li P , He C Y , Xu Q , et al. Effect of the-2081G/A Polymorphism of the TLR4 Gene and Its Interaction with Helicobacter pylori Infection on the Risk of Gastric Cancer in Chinese Individuals[J]. Genetic Testing and Molecular Biomarkers, 2014, 18(9).

393. Qiong L, Jun Y, Xu W, et al. B-cell Lymphoma 2 rs17757541 C>G polymorphism was associated with an increased risk of gastric cardiac adenocarcinoma in a Chinese population[J]. Asian Pacific Journal of Cancer Prevention Apjcp, 2013, 14(7):4301-4306.

394. Guo B W , Yang L , Zhao R , et al. Association between ERCC5 gene polymorphisms and gastric cancer risk[J]. Genetics and molecular research: GMR, 2016, 15(2).

395. Li T , Cao B W , Dai Y , et al. Correlation of transforming growth factor beta-1 gene polymorphisms C-509T and T869C and the risk of gastric cancer in China[J]. Journal of Gastroenterology and Hepatology, 2008, 23(4):638-642.

396. Li T , Qin W , Liu Y , et al. Effect ofRAGEgene polymorphisms and circulating sRAGE levels on susceptibility to gastric cancer: a case–control study[J]. Cancer Cell International, 2017, 17(1):19.

397. Li W Q , Hu N , Hyland P L , et al. Genetic variants in DNA repair pathway genes and risk of esophageal squamous cell carcinoma and gastric adenocarcinoma in a Chinese population.[J]. Carcinogenesis, 2013, 34(7):1536-1542.

398. Li X , Yang X X , Hu N Y , et al. A risk-associated single nucleotide polymorphism ofSMAD7is common to colorectal, gastric, and lung cancers in a Han Chinese population[J]. Molecular Biology Reports, 2011, 38(8):5093-5097.

399. Li Y , He W , Liu T , et al. A New Cyclo-Oxygenase-2 Gene Variant in the Han Chinese Population is Associated with an Increased Risk of Gastric Carcinoma[J]. Molecular Diagnosis & Therapy, 2010, 14(6):351-355.

400. Li Y , Tang Y , Zhou R , et al. Genetic Polymorphism in the 3 '-Untranslated Region of the E-Cadherin Gene Is Associated With Risk of Different Cancers[J]. Molecular Carcinogenesis, 2011, 50 (11):857-862.

401. Li Z H , Pan X M , Han B W , et al. A let-7 binding site polymorphism rs712 in theKRAS3′ UTR is associated with an increased risk of gastric cancer[J]. Tumor Biology, 2013, 34(5):3159-3163.

402. Li Z Q , Yu W P , Xie X D , et al. Association of gastric cancer with tyrosine hydroxylase gene polymorphism in a northwestern Chinese population[J]. Clinical & Experimental Medicine, 2007, 7(3):98-101.

403. Li Z , Chen D , Zhang C , et al. HLA polymorphisms are associated withHelicobacter pyloriinfected gastric cancer in a high risk population, China[J]. Immunogenetics, 2005, 56(11):781-787.

404. Lin S H , Li Y H , Leung K , et al. Salt Processed Food and Gastric Cancer in a Chinese Population[J]. Asian Pacific journal of cancer prevention: APJCP, 2014, 15(13):5293-5298.

405. Lin X , Hu D , Chen G , et al. Associations of THBS2 and THBS4 polymorphisms to gastric cancer in a Southeast Chinese population[J]. Cancer Genetics, 2016, 209(5):215-222.

406. Lin Y , Nie Y , Zhao J , et al. Genetic polymorphism at miR-181a binding site contributes to gastric cancer susceptibility[J]. Carcinogenesis, 2012, 33(12):2377-2383.

407. Lin Y , Yuan J , Wang L , et al. Correlation between SNPs in CDH1 and gastric cancer in Chinese population[J]. Open Medicine, 2015, 10(1):57-62.

408. Liu, B. and F. Li, et al. (2018). Circulating SH2B1 is associated with an increased risk of gastric cancer. ONCOLOGY LETTERS 15(5): 7305-7311.

409. Liu J W , He C Y , Sun L P , et al. The DNA Repair Gene ERCC6 rs1917799 Polymorphism is Associated with Gastric Cancer Risk in Chinese[J]. Asian Pacific journal of cancer prevention: APJCP, 2013, 14(10):6103-6108.

410. Liu J , Sun L , Xu Q , et al. Association of nucleotide excision repair pathway gene polymorphisms with gastric cancer and atrophic gastritis risks[J]. Oncotarget, 2016, 7(6):6972-6983.

411. Liu K , Lei Z , Yao H , et al. Impact of a Eukaryotic Translation Initiation Factor 3a Polymorphism on Susceptibility to Gastric Cancer[J]. Medical Principles and Practice, 2016, 25(5):461-465.

412. Association of candidate genetic variations with gastric cardia adenocarcinoma in Chinese population: a multiple interaction analysis[J]. Carcinogenesis, 2011, 32(3):336-342.

413. Liu W , Tian T , Liu L , et al. A functional SNP rs1892901 in FOSL1 is associated with gastric cancer in Chinese population[J]. Scientific Reports, 2017, 7:41737.

414. Liu X , Bao G , Huo T , et al. Constitutive telomere length and gastric cancer risk: Case-control analysis in Chinese Han population[J]. 2009, 100(7):1300-1305.

415. Liu Y , Xu J , Jiang M , et al. Association Between Functional PSMD10 Rs111638916 Variant Regulated by MiR-505 and Gastric Cancer Risk in a Chinese Population[J]. Cellular Physiology and Biochemistry, 2015, 37(3):1010-1017.

416. A germline variant N375S in MET and gastric cancer, susceptibility in a Chinese population[J]. Journal of Biomedical Research 26(5): 315-318.

417. Liu Y , Lei T , Zhang N , et al. Leukocyte telomere length and risk of gastric cardia adenocarcinoma[J]. Scientific Reports, 2018, 8(1).

418. Liu Z, Shi Y, Na Y, et al. Genetic polymorphisms in TNIP1 increase the risk of gastric carcinoma[J]. Oncotarget, 2016, 7(26):40500-40507.

419. Lu J J , Zhang H Q , Mai P , et al. Lack of association between ERCC5 gene polymorphisms and gastric cancer risk in a Chinese population[J]. Genetics and Molecular Research, 2016, 15(2).

420. Du M , Wang W , Jin H , et al. The association analysis of lncRNA HOTAIR genetic variants and gastric cancer risk in a Chinese population[J]. Oncotarget, 2015, 6(31).

421. Lu R , Gao X , Chen Y , et al. Association of an NFKB1 intron SNP (rs4648068) with gastric cancer patients in the Han Chinese population[J]. BMC Gastroenterology, 2012, 12(1):87.

422. Liu K , Lei Z , Yao H , et al. Impact of a Eukaryotic Translation Initiation Factor 3a Polymorphism on Susceptibility to Gastric Cancer[J]. Medical Principles and Practice, 2016, 25(5):461-465.

423. Lu, X, Wang X, Liu L, et al. (2016). ERCC2 Lys751Gln genetic variation is associated with the susceptibility to gastric cancer in a Chinese population. INTERNATIONAL JOURNAL OF CLINICAL AND EXPERIMENTAL PATHOLOGY 9(3): 3912-3918.

424. Lu Y , Chen J , Ding Y , et al. Genetic variation of PSCA gene is associated with the risk of both diffuse- and intestinal-type gastric cancer in a Chinese population[J]. International Journal of Cancer, 2010, 127(9):2183-2189.

425. YanLu, Yao-Chuxu, JingShen, et al. E-cadherin gene C-160A promoter polymorphism and risk of non-cardia gastric cancer in a Chinese population[J]. World J Gastroenterol 11(1): 56-60.

426. Lu Z , Luo T , Nie M , et al. Genetic polymorphisms of XRCC1 gene and susceptibility to gastric cancer in Chinese Han population[J]. Biomarkers, 2013, 18(6).

427. Luo D, Wang Y, Huan X, et al. Identification of a synonymous variant in TRIM59 gene for gastric cancer risk in a Chinese population[J]. Oncotarget, 2017, 8(7):11507-11516.

428. Ying L , Sihong Y , Jirong W , et al. Association between Sumoylation-Related Gene rs77447679 Polymorphism and Risk of Gastric Cancer (GC) in a Chinese Population[J]. Journal of Cancer, 2017, 8(16):3226-3231.

429. Lv Z , Sun L , Xu Q , et al. Long non-coding RNA polymorphisms in 6p21.1 are associated with atrophic gastritis risk and gastric cancer prognosis[J]. Oncotarget, 2017, 8(56):95303-95315.

430. Ma X, Huang C, Luo D, et al. Tag SNPs of long non-coding RNA TINCR affect the genetic susceptibility to gastric cancer in a Chinese population[J]. Oncotarget, 2016, 7(52):87114.

431. Ma X , Yang C , Tang R , et al. Association between LMP2 and LMP7 gene polymorphisms and the risk of gastric cancer: A case-control study[J]. Oncology letters, 2015, 10(1)..

432. Mao H , Cui R , Wang X . Association analysis of selenoprotein S polymorphisms in Chinese Han with susceptibility to gastric cancer[J]. International Journal of Clinical & Experimental Medicine, 2015, 8(7):10993.

433. Meng H , Lu S , Zhang Z , et al. Association of XRCC1 gene polymorphisms with susceptibility to gastric cancer in Chinese Han population[J]. Journal of Pharmacy & Pharmacology, 2015, 66(10):1463-1468.

434. Miao X , Xing D , Tan W , et al. Susceptibility to gastric cardia adenocarcinoma and genetic polymorphisms in methylenetetrahydrofolate reductase in an at-risk

Chinese population.[J]. Cancer Epidemiology Biomarkers & Prevention, 2002, 11(11):1454-1458.

435. Miao X , Yu C , Tan W , et al. A functional polymorphism in the matrix metalloproteinase-2 gene promoter (-1306C/T) is associated with risk of development but not metastasis of gastric cardia adenocarcinoma[J]. Cancer Research, 2003, 63(14):3987-3990.

436. Miao X , Zhang X , Zhang L , et al. Adenosine Diphosphate Ribosyl Transferase and X-Ray Repair Cross-Complementing 1 Polymorphisms in Gastric Cardia Cancer[J]. Gastroenterology, 2006, 131(2):420-427.

437. Mou X, Li T, Wang J, et al. Genetic Variation of BCL2 (rs2279115), NEIL2 (rs804270), LTA (rs909253), PSCA (rs2294008) and PLCE1 (rs3765524, rs10509670) Genes and Their Correlation to Gastric Cancer Risk Based on Universal Tagged Arrays and Fe3O4 Magnetic Nanoparticles[J]. Journal of Biomedical Nanotechnology, 2015, 11(11): 2057-2066.

438. Mu L N , Cao W , Zhang Z F , et al. Polymorphisms of 5,10-methylenetetralydrofolate reductase (MTHFR), fruit and vegetable intake, and the risk of stomach cancer[J]. Biomarkers, 2007, 12(1):61-75.

439. Mu L N , Lu Q Y , Yu S Z , et al. Green tea drinking and multigenetic index on the risk of stomach cancer in a Chinese population[J]. International Journal of Cancer, 2005, 116(6):972-983.

440. Mu Y P , Su X L . Polymorphism in pre-miR-30c contributes to gastric cancer risk in a Chinese population[J]. Medical Oncology, 2011, 29(3):1723-1732.

441. Pan X F , Xie Y , Loh M , et al. Polymorphisms of XRCC1 and ADPRT Genes and Risk of Noncardia Gastric Cancer in a Chinese Population: a Case-control Study[J]. Asian Pacific journal of cancer prevention: APJCP, 2012, 13(11):5637-5642. 442. Pan X F , Yang S J , Loh M , et al. Interleukin-10 gene promoter polymorphisms and risk of gastric cancer in a Chinese population: Single nucleotide and haplotype analyses[J]. Asian Pac J Cancer Prev, 2013, 14(4):2577-2582.

443. Pan X M, Sun R F, Li Z H, et al. Pri-miR-34b/c rs4938723 Polymorphism Is Associated with a Decreased Risk of Gastric Cancer[J]. Genetic Testing & Molecular Biomarkers, 2015, 19(4):198-202.

444. Pan, X. and Y. Li, et al. (2013). Pan X , Li Y , Feng J , et al. A functional polymorphism T309G in MDM2 gene promoter, intensified by Helicobacter pylori lipopolysaccharide, is associated with both an increased susceptibility and poor prognosis of gastric carcinoma in Chinese patients[J]. BMC Cancer, 2013, 13: 126.

445. Peng S , Kuang Z , Sheng C , et al. Association of MicroRNA-196a-2 Gene Polymorphism with Gastric Cancer Risk in a Chinese Population[J]. Digestive Diseases and Sciences, 2010, 55(8):2288-2293.

446. Qi L , Sun K , Zhuang Y , et al. Study on the association between PI3K/AKT/mTOR signaling pathway gene polymorphism and susceptibility to gastric cancer[J]. Journal of B.U.ON.: official journal of the Balkan Union of Oncology, 2017, 22(6):1488-1493.

447. Qi W T , Gao J L , Zhang S S . Role of IL-17 gene polymorphisms in the susceptibility to gastric cancer.[J]. Genetics & Molecular Research Gmr, 2015, 14(4):13364.

448. Qiao W , Wang T , Zhang L , et al. Association Study of Single Nucleotide Polymorphisms in, XRCC1, Gene with the Risk of Gastric Cancer in Chinese Population[J]. International Journal of Biological Sciences, 2013, 9(7):753-758.

449. Qiao W , Wang T , Zhang L , et al. Association between single genetic polymorphisms ofMDR1gene and gastric cancer susceptibility in Chinese[J]. MEDICAL ONCOLOGY, 2013, 30(3):643.

450. Qin S Y , Yang X W , Luo W , et al. Association of interleukin 22 polymorphisms with gastric cancer risk[J]. Tumor Biology, 2015, 36(3):2033-2039. 451. Qinghai Z , Yanying W , Yunfang C , et al. Effect of interleukin-17A and interleukin-17F gene polymorphisms on the risk of gastric cancer in a Chinese population[J]. Gene, 2013, 537(2):328-332.

452. Jiong-Liang, Qiu, Jian-Ning, et al. Nutritional factors and gastric cancer in Zhoushan Islands, China[J]. World Journal of Gastroenterology, 2005, 11(28):4311-4316.

453. Qiu L X , He J , Cheng L , et al. Genetic variant of PRKAA1 and gastric cancer risk in an Eastern Chinese population[J]. Oncotarget, 2015, 6(40):42661-42666.

454. Qiu L X , Hua R X , Cheng L , et al. Genetic variant rs4072037 of MUC1 and gastric cancer risk in an Eastern Chinese population[J]. Oncotarget, 2016, 7(13).

455. Ren Z , Li M , Liu R , et al. Interleukin 17A rs3819024 A>G polymorphism is associated with an increased risk of gastric cardia adenocarcinoma in a Chinese population[J]. Biomarkers, 2014, 19(5):411-416.

456. Sang L , Lv Z , Sun L P , et al. Impact of SNP-SNP interactions of DNA repair gene ERCC5 and metabolic gene GSTP1 on gastric cancer/atrophic gastritis risk in a Chinese population[J]. World Journal of Gastroenterology, 2018(5): 602-612.

457. Savage S A , Abnet C C , Haque K , et al. Polymorphisms in interleukin -2, -6, and -10 are not associated with gastric cardia or esophageal cancer in a high-risk chinese population[J]. Cancer Epidemiol Biomarkers Prev, 2004, 13(9):1547-1549.

458. Setiawan V W , Yu G P , Lu Q Y , et al. Allium vegetables and stomach cancer risk in China.[J]. Asian Pac J Cancer Prev, 2005, 6(3):387-395.

459. Setiawan V W , Zhang Z F , Yu G P , et al. GSTT1 and GSTM1 null genotypes and the risk of gastric cancer: A case- control study in a Chinese population[J]. Cancer Epidemiology Biomarkers & Prevention, 2000, 9(1):73-80.

460. Setiawan V W, Zhang Z F, Yu G P, et al. GSTP1 polymorphisms and gastric cancer in a high-risk Chinese population.[J]. Cancer Causes & Control, 2001, 12(8):673-681.

461. Setiawan V W , Zhang Z F , Yu G P , et al. Protective effect of green tea on the risks of chronic gastritis and stomach cancer[J]. International journal of cancer. Journal international du cancer, 2001, 92(4):600-604.

462. Shao A , Zheng L , Chen S , et al. p21, p53, TP53BP1 and p73 polymorphisms and the risk of gastric cardia adenocarcinoma in a Chinese population[J]. Biomarkers, 2015.

463. Shen H, Newmann AS, Hu Z, Zhang Z, et al. Methylenetetrahydrofolate reductase polymorphisms/haplotypes and risk of gastric cancer: a case-control analysis in China. Oncol Rep, 2005, 13(2): 355-60.

464. Shen H, Solari A, Wang X, et al. P53 codon 72 polymorphism and risk of gastric cancer in a Chinese population. Oncol Rep, 2004, 11(5): 1115-20.

465. Shen H , Wang X , Hu Z , et al. Polymorphisms of DNA repair gene XRCC3 Thr241Met and risk of gastric cancer in a Chinese population[J]. Cancer Letters, 2004, 206(1):0-58.

466. Shen J , Wang R T , Xu Y C , et al. Interaction models of CYP1A1, GSTMl polymorphisms and tobacco smoking in intestinal gastric cancer[J]. World Journal of Gastroenterology, 2005, 11(38):6056-6060.

467. Shen X , Zhang J , Yan Y , et al. Analysis and Estimates of the Attributable Risk for Environmental and Genetic Risk Factors in Gastric Cancer in a Chinese Population[J]. Journal of Toxicology and Environmental Health, Part A, 2009, 72(11-12):759-766.

468. Shen YY, Lu YC, Shen DP, et al. Fibroblast growth factor receptor 4 Gly388Arg polymorphism in Chinese gastric cancer patients. World J Gastroenterol, 2013, 19(28): 4568-75.

469. Shi J , Liu Y , Liu J , et al. Hsa-miR-449a genetic variant is associated with risk of gastric cancer in a Chinese population[J]. International journal of clinical and experimental pathology, 2015, 8(10):13387-13392.

470. Shi Y , Chen X , Xi B , et al. SNP rs3202538 in 3’UTR region of ErbB3 regulated by miR-204 and miR-211 promote gastric cancer development in Chinese population[J]. Cancer Cell International, 2017, 17(1):81.

471. Shi Y , Hu Z , Wu C , et al. A genome-wide association study identifies new susceptibility loci for non-cardia gastric cancer at 3q13.31 and 5p13.1[J]. Nature Genetics.

472. Song B , Yan G , Hao H , et al. rs11671784 G/A and rs895819 A/G Polymorphisms Inversely Affect Gastric Cancer Susceptibility and miR-27a Expression in a Chinese Population[J]. Medical Science Monitor International Medical Journal of Experimental & Clinical Research, 2014, 20(20):2318-2326.

473. Song Q , Zhang Z , Liu Y , et al. The tag SNP rs10746463 in decay-accelerating factor is associated with the susceptibility to gastric cancer[J]. Molecular Immunology, 2015, 63(2):473-478.

474. Song Q , Hu P , Wang J , et al. Association between gastric cardia adenocarcinoma risk and alcohol flushing response, but not alcohol consumption[J]. Medical Oncology, 2014, 31(3):858-74.

475. Yong-Xi S , Xin Z , Zhen-Ning W , et al. The Association between Individual SNPs or Haplotypes of Matrix Metalloproteinase 1 and Gastric Cancer Susceptibility, Progression and Prognosis[J]. PLoS ONE, 2012, 7(5):e38002.

476. Stolzenberg-Solomon R Z , Qiao Y L , Abnet C C , et al. Esophageal and Gastric Cardia Cancer Risk and Folate- and Vitamin B12-related Polymorphisms in Linxian, China[J]. Cancer Epidemiology Biomarkers & Prevention, 2003, 12(11 Pt 1):1222-1226.

477. Su R, Li, WX, Luo RC. Association between miR-146a, miR-149, miR-196a2 and miR-499 gene polymorphisms and the susceptibility to gastric cancer in a Chinese population. INTERNATIONAL JOURNAL OF CLINICAL AND EXPERIMENTAL PATHOLOGY, 2016, 9(2): 2192-2199.

478. Sun H , Wu X , Wu F , et al. Associations of Genetic Variants in the PSCA, MUC1 and PLCE1 Genes with Stomach Cancer Susceptibility in a Chinese Population[J]. PLOS ONE, 2015, 10.

479. Sun L M , Shang Y , Zeng Y M , et al. HOGG1 polymorphism in atrophic gastritis and gastric cancer after Helicobacter pylori eradication[J]. World Journal of Gastroenterology, 2010, 16(35):4476.

480. Sun P , Du J , Zhu X , et al. Genetic Variation in the 3'-Untranslated Region of NBN Gene Is Associated with Gastric Cancer Risk in a Chinese Population[J]. PLOS ONE, 2015, 10(9).

481. Sun Q , Gu H , Zeng Y , et al. Hsa-mir-27a genetic variant contributes to gastric cancer susceptibility through affecting miR-27a and target gene expression[J]. Cancer science, 2010, 101(10):2241-2247.

482. Dietary Protective and Risk Factors for Esophageal and Stomach Cancers in a Low-epidemic Area for Stomach Cancer in Jiangsu Province, China: Comparison with Those in a High-epidemic Area[J]. Cancer Science, 2001, 92(11):1157-1165. 483. Takezaki T , Gao C M , Wu J Z , et al. HOGG1 Ser326Cys polymorphism and modification by environmental factors of stomach cancer risk in Chinese[J]. International Journal of Cancer, 2002, 99(4):624-627.

484. Tan, W. Significant increase in risk of gastroesophageal cancer is associated with interaction between promoter polymorphisms in thymidylate synthase and serum folate status[J]. Carcinogenesis, 2005, 26(8):1430-1435.

485. Tang W Y , Wang L , Li C , et al. Identification and Functional Characterization of\r, JWA\r, Polymorphisms and their Association with Risk of Gastric Cancer and Esophageal Squamous Cell Carcinoma in a Chinese Population[J]. Journal of Toxicology and Environmental Health, Part A, 2007, 70(11):885-894.

486. Tang, W, Wang Y, Chen J, et al. Investigation of Cytotoxic T-lymphocyte antigen 4 Polymorphisms in Gastric Cardia Adenocarcinoma. Scand J Immunol, 2016, 83(3): 212-8.

487. Tarleton, HP, Chang, SC, Park, SL et al. Genetic variation at 8q24, family history of cancer, and upper gastrointestinal cancers in a Chinese population. Fam Cancer, 2014, 13(1): 45-56.

488. Tian T , Xiao L , Du J , et al. Polymorphisms in CARS are associated with gastric cancer risk: a two-stage case–control study in the Chinese population[J]. Gastric Cancer, 2017, 20(6): 940-947.

489. Wang B , Yang H , Shen L , et al. Rs56288038 (C/G) in 3\"UTR of IRF-1 Regulated by MiR-502-5p Promotes Gastric Cancer Development[J]. Cellular Physiology and Biochemistry, 2016, 40(1-2):391-399.

490. Wang C, Xu S, Yi F, et al. Tumor Necrosis Factor-Related Apoptosis Inducing Ligand Gene Polymorphisms are Correlated with Gastric Cancer in Central China[J]. Pharmaceutical Research, 2015, 32(3):762-768.

491. Genetic polymorphism in chemokine CCL22 and susceptibility to Helicobacter pylori infection-related gastric carcinoma[J]. Cancer, 2009, 115(11): 2430-7.

4892. Wang J Y , Yang I P , Wu D C , et al. Functional glutathione peroxidase 3 polymorphisms associated with increased risk of Taiwanese patients with gastric cancer[J]. Clinica Chimica Acta, 2010, 411(19-20):0-1436.

493. Wang J , Zhang J , Zhou C , et al. An Insertion/Deletion Polymorphism Within the Proximal Promoter of\r, EGLN2\r, Is Associated With Susceptibility for Gastric Cancer in the Chinese Population[J]. Genetic Testing and Molecular Biomarkers, 2014, 18(4):269-273.

494. Wang K , Xu L , Pan L , et al. The functionalBRCA1rs799917 genetic polymorphism is associated with gastric cancer risk in a Chinese Han population[J]. Tumor Biology, 2015, 36(1):393-397.

495. Wang L , Chen W , Wang J , et al. Reduced folate carrier gene G80A polymorphism is associated with an increased risk of gastroesophageal cancers in a chinese population[J]. European Journal of Cancer, 2006, 42(18):0-3211.

496. Wang L , Ke Q , Chen W , et al. Polymorphisms of MTHFD, Plasma Homocysteine Levels, and Risk of Gastric Cancer in a High-Risk Chinese Population[J]. Clinical Cancer Research, 2007, 13(8):2526-2532.

497. Wang, M. Y. and J. He, et al. (2016). Wang M Y , He J , Zhu M L , et al. A Functional Polymorphism (rs2494752) in the AKT1 Promoter Region and Gastric Adenocarcinoma Risk in an Eastern Chinese Population[J]. Scientific Reports, 2015, 6: 20008.

498. Wang M Y , Jia M , He J , et al. MDM4 genetic variants and risk of gastric cancer in an Eastern Chinese population[J]. Oncotarget, 2016, 8(12): 19547-19555.

499. Wang M Y , Li Q X , He J , et al. Genetic variations in the mTOR gene contribute to gastric adenocarcinoma susceptibility in an Eastern Chinese population[J]. Pharmacogenetics and Genomics, 2015, 25(11):521-530.

500. Meng-Yun W , Mei-Ling Z , Jing H , et al. Potentially Functional Polymorphisms in the CASP7 Gene Contribute to Gastric Adenocarcinoma Susceptibility in an Eastern Chinese Population[J]. PLoS ONE, 2013, 8(9):e74041. 501. Wang M , Wu D , Tan M , et al. FASandFASLigand Polymorphisms in the Promoter Regions and Risk of Gastric Cancer in Southern China[J]. Biochemical Genetics, 2009, 47(7-8):559-568.

502. Wang M , Zhang R , He J , et al. Potentially functional variants of PLCE1 identified by GWASs contribute to gastric adenocarcinoma susceptibility in an eastern Chinese population.[J]. Plos One, 1932, 7(3):e31932.

503. Wang N , Qiao Q , Bao G , et al. Genetic polymorphisms are associated with the risk of gastric and colorectal cancers in a Han Chinese population[J]. Oncotarget, 2017, 8(17):28805-28811.

504. Wang N , Yang J , Lu J , et al. IL-17 gene polymorphism is associated with susceptibility to gastric cancer[J]. Tumor Biology, 2014, 35(10):10025-10030.

505. Association of NOD1 and NOD2 genes polymorphisms with Helicobacter pylori related gastric cancer in a Chinese population[J]. World J Gastroenterol, 2012, 18(17): 2112-20.

506. Wang S , Tian L , Zeng Z , et al. IkappaBalpha polymorphism at promoter region (rs2233408) influences

the susceptibility of gastric cancer in Chinese.[J]. Bmc Gastroenterology, 2010, 10(1):1-6.

507. Wang S , Zhang M , Zeng Z , et al. IκBα polymorphisms were associated with increased risk of gastric cancer in a southern Chinese population: A case–control study[J]. Life Sciences, 2011, 88(17-18): 792-797.

508. Wang S , Tian Y , Wu D , et al. Genetic variation of CTNNB1 gene is associated with susceptibility and prognosis of gastric cancer in a Chinese population[J]. Mutagenesis, 2012, 27(6):623-630.

509. Wang W , Li F , Mao Y , et al. A miR-570 binding site polymorphism in theB7-H1gene is associated with the risk of gastric adenocarcinoma[J]. Human Genetics, 2013, 132(6):641-648.

510. Wang, X, Dong,XQ, Yin J, et al. Tagging polymorphisms of methyl-CpG binding domain 4 and gastric cardiac adenocarcinoma risk in a Chinese population. Dis Esophagus, 2017, 30(2): 1-6.

511. Wang X , Li T , Li M , et al. The Functional SOCS3 RS115785973 Variant Regulated by MiR-4308 Promotes Gastric Cancer Development in Chinese Population[J]. Cellular Physiology and Biochemistry, 2016, 38(5):1796-1802.

512. Wang X , Yang J , Ho B , et al. Interaction of Helicobacter pylori with Genetic Variants in the MDM2 Promoter, is Associated with Gastric Cancer Susceptibility in Chinese Patients[J]. Helicobacter, 2009, 14(5):466-471.

513. Wang X , Yin J , Zheng L , et al. The variant interleukin 1f7 rs3811047 G>A was associated with a decreased risk of gastric cardiac adenocarcinoma in a Chinese Han population[J]. Tumor Biology, 2014, 35(4):3509-3515.

514. Wang YM, Wang R, Wen D, et al. Single nucleotide polymorphism in DNA methyltransferase 3B promoter and its association with gastric cardiac adenocarcinoma in North China. World J Gastroenterol, 2005, 11(23): 3623-3627.

515. Wang, YF. and Chen SC, Kang MQ, et al. Genetic variations in MTHFR and gastric cardia adenocarcinoma susceptibility in the Chinese Han population. International Journal of Clinical and Experimental Medicine, 2015, 8(10): 18936-18944.

516. Wang Y , Li H , Wang X , et al. Association between four SNPs in IL-4 and the risk of gastric cancer in a Chinese population[J]. International Journal of Molecular Epidemiology & Genetics, 2017, 8(4): 45-52.

517. Wei L , Zhao Y , Guo T K , et al. Association of mtDNA D-Loop Polymorphisms with Risk of Gastric Cancer in Chinese Population[J]. Pathology Oncology Research Por, 2011, 17(3):735-742.

518. Wu C , Wang G , Yang M , et al. Two Genetic Variants in Prostate Stem Cell Antigen and Gastric Cancer Susceptibility in a Chinese Population[J]. Molecular Carcinogenesis, 2009, 48(12):1131-1138.

519. Wu D M, Zhu HX, Zhao QH, et al. Genetic variations in the SMAD4 gene and gastric cancer susceptibility[J]. World Journal of Gastroenterology, 2010, 16(44):5635-5641.

520. Wu D , Tian Y , Gong W , et al. Genetic variants in the Runt-related transcription factor 3 gene contribute to gastric cancer risk in a Chinese population[J]. Cancer Science, 2009, 100.

521. Wu GC , Zhang ZT . Genetic association of single nucleotide polymorphisms in P53 pathway with gastric cancer risk in a Chinese Han population[J]. Medical Oncology, 2015, 32(1):401.

522. Wu H , Gu Y , Wei L , et al. Association of Romo1 Gene Genetic Polymorphisms with Risk of Gastric Cancer in Northwestern Chinese Population[J]. PATHOLOGY & ONCOLOGY RESEARCH, 2015, 21(3):581-587.

523. Wu H , Wang Y , Wang S , et al. Is susceptibility locus for lung cancer in the 15q25 nicotinic acetylcholine receptor gene cluster CHRNA5-A3-B4 associated with risk of gastric cancer?[J]. Medical Oncology, 2013, 30(2):576.

524. Huazhang W , Kun Z , Pihai G , et al. A Novel Functional TagSNP Rs7560488 in the DNMT3A1 Promoter Is Associated with Susceptibility to Gastric Cancer by Modulating Promoter Activity[J]. PLoS ONE, 2014, 9(3):e92911.

525. Wu J , Lu Y , Ding Y B , et al. Promoter polymorphisms of IL2, IL4, and risk of gastric cancer in a high-risk Chinese population[J]. Molecular carcinogenesis, 2009, 48(7):626-632.

526. Wu J , Tong S , Zhan Z , et al. TGF-α gene variations and increased susceptibility of gastric cancer in an eastern Chinese Han population[J]. Biomarkers, 2014:19(1): 9-15.

527. Juan W , Junfeng Z , Qinhong C , et al. Genetic Variants in the 3′ Untranslated Region of sFRP1 Gene and Risk of Gastric Cancer in a Chinese Population[J]. The International Journal of Biological Markers, 2017, 32(1):102-107.

528. Wu, MS. and Huang SP, Chang YT, et al. Association of the - 160 C → A promoter polymorphism of E-cadherin gene with gastric carcinoma risk. Cancer, 2002, 94(5): 1443-1448.

529. Wu Q , Lu S , Wang L , et al. DNMT3A rs36012910 A>G polymorphism and gastric cancer susceptibility in a Chinese population[J]. Molecular Biology Reports, 2012, 39(12):10949-10955.

530. Wu R , Li F , Zhu J , et al. A functional variant at miR-132-3p, miR-212-3p, and miR-361-5p binding site inCD80gene alters susceptibility to gastric cancer in a Chinese Han population[J]. Medical Oncology, 2014, 31(8).

531. Wu X J , Mi Y Y , Yang H , et al. Association of the hsa-mir-499 (rs3746444) Polymorphisms with Gastric Cancer Risk in the Chinese Population[J]. Onkologie, 2013, 36(10):573-576.

532. Wu X , Zeng Z , Chen B , et al. Association between polymorphisms in interleukin-17A and interleukin-17F genes and risks of gastric cancer[J]. International Journal of Cancer, 2010, 127(1):86-92.

533. Xia, ZG, Yin HF, Long Y, et al. Genetic variant of miR-146a rs2910164 C>G and gastric cancer susceptibility. Oncotarget, 2016, 7(23): 34316-21.

534. Xiang F , Ni Z , Zhan Y , et al. Association of 758 G/A polymorphism of 3'untranslated region of prohibitin with risk of gastric cancer[J]. Journal of Clinical Laboratory Analysis, 2017:e22182.

535. Xu B L , Li Y T , Dong S X , et al. IL-17 rs2275913 genetic variation contributes to the development of gastric cancer in a Chinese population[J]. Genetics and molecular research: GMR, 2016, 15(2).

536. Xu, L and Zeng, ZR, Chen B, et al.. Association between the TGFB1 -509C/T and TGFBR2 -875A/G polymorphisms and gastric cancer: A case-control study. Oncology Letters, 2011, 2(2): 371-377.

537. Xu M, Qiang F, Gao Y, et al. Evaluation of a Novel Functional Single-Nucleotide Polymorphism (rs35010275 G>C) in MIR196A2 Promoter Region as a Risk Factor of Gastric Cancer in a Chinese Population[J]. Medicine, 2014, 93(26):e173.

538. Qian X , Jing-Wei L , Cai-Yun H , et al. The Interaction Effects of pri-let-7a-1 rs10739971 with PGC and ERCC6 Gene Polymorphisms in Gastric Cancer and Atrophic Gastritis[J]. PLoS ONE, 2014, 9(2):e89203.

539. Qian X, Wu Y F, Ying L, et al. SNP-SNP interactions of three new pri-miRNAs with the target genePGCand multidimensional analysis ofH. pyloriin the gastric cancer/atrophic gastritis risk in a Chinese population[J]. Oncotarget, 2016, 7(17):23700-23714.

540. Xu R , Peng C , Xiao S , et al. IFRD1 polymorphisms and gastric cancer risk in a Chinese population[J]. Medical Oncology, 2014, 31(9).

541. Xu S , Zhou Y , Du W D , et al. Association of the variant rs2243421 of human DOC-2/DAB2 interactive protein gene (hDAB2IP) with gastric cancer in the Chinese Han population[J]. Gene, 2013, 515(1):200-204.

542. Xu T , Fu D , Ren Y , et al. Genetic variations of TLR5 gene interacted with Helicobacter pylori infection among carcinogenesis of gastric cancer[J]. Oncotarget, 2017, 8(19):31016-31022.

543. Xu Y , Cao X , Jiang J , et al. TNF-α-308/-238 polymorphisms are associated with gastric cancer: A case-control family study in China[J]. Clinics and Research in Hepatology and Gastroenterology, 2016, 41(1): 103-109.

544. Yan L , Yanan D , Donglan S , et al. Polymorphisms of XRCC1 gene and risk of gastric cardiac adenocarcinoma[J]. Diseases of the Esophagus, 2009, 22(5):396-401.

545. Yang C , Ma X , Liu D , et al. Promoter polymorphisms of miR-34b/c are associated with risk of gastric cancer in a Chinese population[J]. Tumor Biology, 2014, 35(12):12545-12554.

546. Yang C , Tang R , Ma X , et al. Tag SNPs in long non-coding RNA H19 contribute to susceptibility to gastric cancer in the Chinese Han population[J]. Oncotarget, 2015, 6(17).

547. Yang G , Rao L , Tian L , et al. An Association between EGF and EGFR Gene Polymorphisms with Gastric Cancer in a Chinese Han Population[J]. Hepato-gastroenterology, 2012, 59(120):2668.

548. Yang J , Hu Z , Xu Y , et al. Interleukin-1B gene promoter variants are associated with an increased risk of gastric cancer in a Chinese population[J]. Cancer Letters, 2004, 215(2):0-198.

549. Yang L J , Gao W , Bai J Y , et al. Correlation between Interleukin-17 gene polymorphism and gastric cancer susceptibility in Han Chinese population[J]. European Review for Medical & Pharmacological Sciences, 2016, 20(7):1271.

550. Yang L Q , Zhang Y , Sun H F . Investigation on ERCC5 genetic polymorphisms and the development of gastric cancer in a Chinese population[J]. Genetics and molecular research: GMR, 2016, 15(3).

551. Yang L , Gu H J , Zhu H J , et al. Tissue inhibitor of metalloproteinase-2 G-418C polymorphism is associated with an increased risk of gastric cancer in a Chinese population[J]. Eur J Surg Oncol, 2008, 34(6):0-641.

552. Yang L , Liu D , Liang S , et al. Janus Kinase 2 Polymorphisms Are Associated with Risk in Patients with Gastric Cancer in a Chinese Population[J]. PLOS ONE, 2013, 8.

553. Yang L , Sun M J , Liu J W , et al. IL-6-6331 (T/C, rs10499563) is Associated with Decreased Risk of Gastric Cancer in Northern Chinese[J]. Asian Pacific journal of cancer prevention: APJCP, 2013, 14(12):7467-7472.

554. Yang L , Zhu H , Zhou B , et al. The Association Between theSurvivinC-31G Polymorphism and Gastric Cancer Risk in a Chinese Population[J]. Digestive Diseases and Sciences, 2009, 54(5):1021-1028.

555. Yang M , Guo Y , Zhang X , et al. Interaction of P53 Arg72Pro and MDM2 T309G polymorphisms and their associations with risk of gastric cardia cancer[J]. Carcinogenesis, 2007, 28(9):1996-2001.

556. Yang W G , Zhang S F , Chen J W , et al. SNPs of Excision Repair Cross Complementing Group 5 and Gastric Cancer Risk in Chinese Populations[J]. Asian Pacific Journal of Cancer Prevention Apjcp, 2012, 13(12):6269.

557. Yin J , Liu C , Wang X , et al. Interleukin15 receptor alphars2228059 A > C polymorphism decreased risk of gastric cardiac adenocarcinoma in a Chinese population[J]. Tumor Biology, 2014, 35(7):6593-6600.

558. Yin J , Pan H , Long T , et al. Polymorphisms of VDR gene and risk of gastric cardiac adenocarcinoma in Chinese population[J]. Oncotarget, 2017, 8(28):45531-45543.

559. Yin J, Wang X, Wei J et al. Interleukin 12B rs3212227 T > G polymorphism was associated with an increased risk of gastric cardiac adenocarcinoma in a Chinese population. Dis Esophagus, 2015, 28(3): 291-8.

560. Yu G P . Green-tea consumption and risk of stomach cancer : a population-based case-control study in Shanghai, China[J]. Cancer Causes Control, 1995, 6(6):532-538.

561. Yu J , Jia Y , Cheung K , et al. Polymorphisms in interleukin-6-6331 influences the susceptibility of a chinese population to gastric cancer[J]. Cancer Investigation, 2011, 136(8):564-572.

562. Yu J , Zeng Z , Wang S , et al. S1951 IL-1b-511 Polymorphism Is Associated with Increased Risk of Certain Subtypes of Gastric Cancer in Chinese: A Case-Control Study[J]. Gastroenterology, 2009, 136(5):A-300.

563. Yuan J M , Yu M C , Xu W W , et al. Helicobacter pylori infection and risk of gastric cancer in Shanghai, China: updated results based upon a locally developed and validated assay and further follow-up of the cohort[J]. Cancer epidemiology, biomarkers & prevention : a publication of the American Association for Cancer Research, cosponsored by the American Society of Preventive Oncology, 1999, 8(7):621: 624.

564. Yuan K , Liu H , Huang L , et al. rs744166 Polymorphism of the STAT3 Gene Is Associated with Risk of Gastric Cancer in a Chinese Population[J]. Journal of Biomedicine and Biotechnology, 2014, 2014(4):527918.

565. Yuan L J , Jin T B , Yin J K , et al. Polymorphisms of tumor-related genes IL-10, PSCA, MTRR and NOC3L are associated with the risk of gastric cancer in the Chinese Han population[J]. Cancer epidemiology, 2012, 36(6).

566. Yuan T , Deng S , Chen M , et al. Association of DNA repair gene XRCC1 and XPD polymorphisms with genetic susceptibility to gastric cancer in a Chinese population[J]. Cancer Epidemiology, 2011, 35(2):170-174.

567. Zeng H M , Pan K F , Zhang Y , et al. Genetic Variants of Toll-Like Receptor 2 and 5, Helicobacter Pylori Infection, and Risk of Gastric Cancer and Its Precursors in a Chinese Population[J]. Cancer Epidemiology Biomarkers & Prevention, 2011, 20(12):2594-2602.

568. Zeng X F, Li J, Li S B. A functional polymorphism in IL-1A gene is associated with a reduced risk of gastric cancer.[J]. Tumor Biology, 2014, 35(1):265-268.

569. Zeng, Ying. Correlation between\r, pre-miR-146a\r, C/G polymorphism and gastric cancer risk in Chinese population[J]. World Journal of Gastroenterology, 2010, 16(28):3578.

570. Zeng Z , Wu X , Chen F , et al. Polymorphisms in prostate stem cell antigen gene rs2294008 increase gastric cancer risk in Chinese[J]. Molecular carcinogenesis, 2011, 50(5):353-358.

571. Zhen Z , Yajun C , Juan W U , et al. Functional epidermal growth factor gene polymorphisms and risk of gastric cancer[J]. Oncology Letters, 2013, 5(2):631-636. 572. Zhan Z , Wu J , Zhang J F , et al. CDH1gene polymorphisms, plasma CDH1 levels and risk of gastric cancer in a Chinese population[J]. Molecular Biology Reports, 2012, 39(8):8107-8113.

573. Zhang A P , Liu B H , Wang L , et al. Glutathione S-transferase gene polymorphisms and risk of gastric cancer in a Chinese population.[J]. Asian Pacific Journal of Cancer Prevention Apjcp, 2011, 12(12):3421.

574. Zhang B , Hao G Y , Gao F , et al. Lack of Association of Common Polymorphisms in MUC1 Gene with H. pylori Infection and Non-cardia Gastric Cancer Risk in a Chinese Population[J]. Asian Pacific journal of cancer prevention: APJCP, 2013, 14(12):7355-7358.

575. Zhang B , Pan K , Liu Z , et al. Genetic polymorphisms of the E-cadherin promoter and risk of sporadic, gastric carcinoma in Chinese populations[J]. Cancer Epidemiol Biomarkers Prev, 2008, 17(9):2402-2408.

576. Zhang C , Ding Z , Lv G , et al. CD226 rs727088A>G polymorphism increases the susceptibility to gastric cancer in Chinese populations[J]. Gene, 2015, 557(1):92-97.

577. Zhang G , Zhang Q Y , Miao X P , et al. Polymorphisms and mutations of the folate receptor-alpha gene and risk of gastric cancer in a Chinese population[J]. International Journal of Molecular Medicine, 2005, 15(4):627.

578. Zhang H , Jin G , Li H , et al. Genetic variants at 1q22 and 10q23 reproducibly associated with gastric cancer susceptibility in a Chinese population[J]. Carcinogenesis, 2011, 32(6):848-852.

579. Zhang, J. Association of the thymidylate synthase polymorphisms with esophageal squamous cell carcinoma and gastric cardiac adenocarcinoma[J]. Carcinogenesis, 2004, 25(12):2479-2485.

580. Zhang J Z , Liu C M , Peng H P , et al. Association of genetic variations in IL-6/IL-6R pathway genes with gastric

cancer risk in a Chinese population[J]. Gene, 2017, 623:1-4.

581. Zhang J , Li Y , Wang R , et al. Association of cyclin D1 (G870A) polymorphism with susceptibility to esophageal and gastric cardiac carcinoma in a northern Chinese population[J]. International Journal of Cancer, 2003, 105(2):281-284.

582. Zhang J , Shi H , Xue M , et al. An insertion/deletion polymorphism in the interleukin-1A 3'untranslated region confers risk for gastric cancer[J]. Cancer biomarkers: section A of Disease markers, 2016, 16(3):359.

583. Junfeng Z , Zhen Z , Juan W , et al. Association among Polymorphisms in EGFR Gene Exons, Lifestyle and Risk of Gastric Cancer with Gender Differences in Chinese Han Subjects[J]. PLoS ONE, 2013, 8(3):e59254.

584. Association between gastric cancer and - 1993 polymorphism of TBX21 gene[J]. World J Gastroenterol 18(10): 1117-22.

585. Association of genotypes of rs671 within ALDH2 with risk for gastric cardia adenocarcinoma in the Chinese Han population in high- and low-incidence areas; Association of genotypes of rs671 within ALDH2 with risk for gastric cardia adenocarcinoma in the Chinese Han population in high- and low-incidence areas; [J]. Cancer Biology & Medicine, 2017, 14(1):60-65.

586. Zhang P , Di J Z , Zhu Z Z , et al. Association of Transforming Growth Factor-beta 1 Polymorphisms with Genetic Susceptibility to TNM Stage I or II Gastric Cancer[J]. Japanese Journal of Clinical Oncology, 2008, 38(12):861-866.

587. Zhang Q , Li Y , Li X , et al. PARP-1 Val762Ala polymorphism, CagA(+) H-pylori infection and risk for gastric cancer in Han Chinese population[J]. MOLECULAR BIOLOGY REPORTS, 2009, 36(6):1461-1467.

588. Variant TP53BP1 rs560191 G〉C is associated with risk of gastric cardia adenocarcinoma in a Chinese Han population[J]. Chin J Cancer Res 27(2): 156-62.

589. Tao Z, Hui W. Variants of interleukin-16 associated with gastric cancer risk[J]. Asian Pacific Journal of Cancer Prevention Apjcp, 2013, 14(9):5269-5273.

590. Zhang WB, Gu HY, Shi YJ, et al. RANK rs1805034 T>C Polymorphism Is Associated with Susceptibility to Gastric Cardia Adenocarcinoma in a Chinese Population. Oncol Res Treat, 2015, 38(10): 503-10.

591. Zhang W , Wang X , Zhou J , et al. Association of interleukin-1B (IL-1B) gene polymorphisms with risk of gastric cancer in Chinese population[J]. CYTOKINE, 2005, 30(6):378-381.

592. Zhang X F , Wang Y M , Ge H , et al. Association of CDH1 single nucleotide polymorphisms with susceptibility to esophageal squamous cell carcinomas and gastric cardia carcinomas[J]. Diseases of the Esophagus, 2008, 21(1): 21-29.

593. Zhang X , Zheng L , Sun Y , et al. Analysis of the association of interleukin-17 gene polymorphisms with gastric cancer risk and interaction withHelicobacter pyloriinfection in a Chinese population[J]. Tumor Biology, 2014, 35(2):1575-1580. 594. Zhang Y , Jin M , Liu B , et al. Association betweenH-RAST81C genetic polymorphism and gastrointestinal cancer risk: A population based case-control study in China[J]. BMC Cancer, 2008, 8(1):256-260.

595. Ye Z , Li-Ping S , Cheng-Zhong X , et al. Interaction between GSTP1 Val Allele and H. pylori Infection, Smoking and Alcohol Consumption and Risk of Gastric Cancer among the Chinese Population[J]. PLoS ONE, 2012, 7(10):e47178.

596. Zhang Z, Xu Y, Zhou J, et al. Polymorphisms of thymidylate synthase in the 5′- and 3′-untranslated regions associated with risk of gastric cancer in South China: a case–control analysis[J]. Carcinogenesis, 2005, 26(10):1764.

597. Zhang Z , Yu D , Lu J , et al. Functional Genetic Variants of TNFSF15 and Their Association with Gastric Adenocarcinoma: A Case-Control Study[J]. PLOS ONE, 2014, 9(9):e108321.

598. Zhao D, Sun T, Zhang X, et al. (2007). Role of CD14 promoter polymorphisms in Helicobacter pylori infection--related gastric carcinoma. Clin Cancer Res 13(8): 2362-8.

599. Zhao J K , Wu M , Kim C H , et al. Jiangsu Four Cancers Study: a large case-control study of lung, liver, stomach, and esophageal cancers in Jiangsu Province, China.[J]. European Journal of Cancer Prevention the Official Journal of the European Cancer Prevention Organisation, 2016, 26(4):357.

600. Zhao L , Wei Y , Song A , et al. Association study between genome-wide significant variants of vitamin B12 metabolism and gastric cancer in a han Chinese population[J]. IUBMB LIFE, 2016, 68(4):303-310.

601. Lina Z , Zhi Z , Jia L , et al. Complement Receptor 1 Genetic Variants Contribute to the Susceptibility to Gastric Cancer in Chinese Population[J]. Journal of Cancer, 2015, 6(6):525-530.

602. Zhao W M , Shayimu P , Liu L , et al. Association between IL-17A and IL-17F gene polymorphisms and risk of gastric cancer in a Chinese population[J]. Genetics and molecular research: GMR, 2016, 15(3).

603. Zhao X M , Chen J , Yang L , et al. Association between IRS-2 G1057D polymorphism and risk of gastric cancer[J]. World Journal of Gastrointestinal Oncology, 2012.

604. Zhi W , Xue B , Wang L , et al. The MLH1 2101C>A (Q701K) variant increases the risk of gastric cancer in Chinese males[J]. BMC Gastroenterology, 2011, 11.

605. Zhou C J , Zhang L W , Gao F , et al. Association Analysis of Common Genetic Variations in MUC5AC Gene with the Risk of Non-cardia Gastric Cancer in a Chinese Population[J]. Asian Pacific Journal of Cancer Prevention Apjcp, 2014, 15(10):4207-10.

606. Zhou C P , Pan H Z , Li F X , et al. Association analysis of colorectal cancer susceptibility variants with gastric cancer in a Chinese Han population[J]. Genetics and Molecular Research, 2014, 13(2):3673-3680.

607. Zhou F , Cheng L , Qiu L X , et al. Associations of potentially functional variants in IL-6, JAKs and STAT3 with gastric cancer risk in an eastern Chinese population[J]. Oncotarget, 2016, 7(19): 28112-23.

608. Zhou F , Qiu L X , Cheng L , et al. Associations of genotypes and haplotypes of IL-17 with risk of gastric cancer in an eastern Chinese population[J]. Oncotarget, 2014, 7(50).

609. Zhou, F, Wang ZB, Zhang l, et al. A functional polymorphism in pre-miR-146a is associated with susceptibility to gastric cancer in a chinese population. DNA and Cell Biology, 2012, 31(7): 1290-1295.

610. Zhou, J. and Z. Wang, et al. (2018). Association of five genetic variations in DNMT1 and DNMT3A with gastric cancer in a Chinese population. Future Oncol 14(17): 1731-1739.

611. Zhou XY, Wang PL, Zhao H. (2018). The association between AURKA gene rs2273535 polymorphism and gastric cancer risk in a Chinese population. Frontiers in Physiology, 2018, 9:1124.

612. Zhou X , Xu L , Yin J . Association between the c.3073A>C genetic polymorphism of the MDR1 gene and susceptibility to gastric cancer in the Chinese Han population.[J]. Genetic Testing & Molecular Biomarkers, 2014, 18(1):66.

613. Zhou Y , Du W D , Chen G , et al. Association analysis of genetic variants in microRNA networks and gastric cancer risk in a Chinese Han population[J]. Journal of Cancer Research & Clinical Oncology, 2012, 138(6):939-945.

614. Zhou Y , Du W D , Wu Q , et al. EZH2 Genetic Variants Affect Risk of Gastric Cancer in the Chinese Han Population[J]. Molecular Carcinogenesis, 2012, 53(8).

615. Zhou Y , Hu W , Zhuang W , et al. Interleukin-10 ?1082 promoter polymorphism and gastric cancer risk in a Chinese Han population[J]. Molecular & Cellular Biochemistry, 2011, 347(1-2):89-93.

616. Zhou Y, Li N, Zhuang W, et al. Vascular endothelial growth factor (VEGF) gene polymorphisms and gastric cancer risk in a Chinese Han population[J]. Molecular Carcinogenesis, 2015, 50(3):184-188.

617. Zhu H , Yang L , Zhou B , et al. Myeloperoxidase G-463A polymorphism and the risk of gastric cancer: a case-control study[J]. Carcinogenesis, 2006, 27(12):2491-2496.

618. Zhu M , Chen X , Zhang H , et al. AluYb8 Insertion in the MUTYH Gene and Risk of Early-onset Breast and Gastric Cancers in the Chinese Population[J]. Asian Pacific journal of cancer prevention: APJCP, 2011, 12(6):1451-1455.

619. He BS, Pan B, Pan YQ, et al. IL-4/IL-4R and IL-6/IL-6R genetic variations and gastric cancer risk in the Chinese population[J]. Am J Transl Res , 2019, 11(6):3698-3706.

620. Wei LS, Niu FG,Wu JM, et al. Association study between genetic polymorphisms in folate metabolism and gastric cancer susceptibility in Chinese Han population: A case–control study[J]. 2019, (5):e633.

621. Liang P, Zhang WT, Wang WH,et al. PLCE1 Polymorphisms and Risk of Esophageal and Gastric Cancer in a Northwestern Chinese Population[J]. Biomed Res Int. 2019:9765191.

622. Liu S, Liu JW, Sun LP,et al. Association of IL10 gene promoter polymorphisms with risks of gastric cancer and atrophic gastritis[J]. J Int Med Res. 2018, 46(12):5155-5166.

623. Liao CW, Hu SQ, et al. Contribution of interaction between genetic variants of interleukin-11 and Helicobacter pylori infection to the susceptibility of gastric cancer[J]. Onco Targets Ther. 2019, 12:7459-7466.

624. Yan KP, Wu K, Lin Chao, et al. Impact of PSCA gene polymorphisms in modulating gastric cancer risk in the Chinese population[J]. Biosci Rep. 2019,39(9):1-7.

625. Hua RX, Zhuo Z, Zhu J,et al. LIG3 gene polymorphisms and risk of gastric cancer in a Southern Chinese population[J]. Gene. 2019, 15;705:90-94.

626. Zhao KX, Zhang R, Li TT, et al. Functional variants of lncRNA LINC00673 and gastric cancer susceptibility: a case-control study in a Chinese population[J]. Cancer Manag Res. 2019, 11:3861-3868.

627. Huang J, Hang JJ, Qin XR,et al. Interaction of H. pylori with toll-like receptor 2-196 to -174 ins/del polymorphism is associated with gastric cancer susceptibility in southern China[J]. Int J Clin Oncol. 2019, 24(5):494-500.

628. Hu D, Liu Q, Lin XD, et al. Association between the receptor for advanced glycation end products gene polymorphisms and cancer risk: a systematic review and meta-analysis[J]. J BUON. 2015, 20(2):614-24.

629.Jia ZF, Cao DH, Wu,YH,et al. Lethal-7-related polymorphisms are associated with susceptibility to and prognosis of gastric cancer[J]. World J Gastroenterol 2019; 25(8): 1012-1023.

630. Tao Y, Mei YX, Ying RB,et al. The ATM rs189037 G>A polymorphism is associated with the risk and prognosis of gastric cancer in Chinese individuals: A case-control study[J]. Gene, 2020, 30,741:144578.

631. Yuan P , Lin L , Zheng KC , et al. Risk factors for gastric cancer and related serological levels in Fujian, China: hospital-based case-control study[J]. BMJ Open, 2020, 10(9):e042341.

632. Zou WJ , Li X , Li C , et al. Analysis of the relationship between MIR155HG variants and gastric Cancer susceptibility[J]. BMC Gastroenterology, 2020, 20(1):17.

633. Kong XL, Yang S, Liu C, et al. Relationship between MEG3 gene polymorphism and risk of gastric cancer in Chinese population with high incidence of gastric cancer[J]. Biosci Rep, 2020, 40(11): BSR20200305.

634. Ma RJ, Qi H. A Variant of Leptin Gene Decreases the Risk of Gastric Cancer in Chinese Individuals: Evidence from a Case-Control Study[J]. Pharmgenomics Pers Med, 2020, 13:397-404.

635. Liu JF, Li HY, Liu YW, et al. MiR-143HG gene polymorphisms as risk factors for gastric cancer in Chinese Han population.[J]. Current molecular medicine, 2020, 20(7):536-547.

636. Bai F, Xiao K. Prediction of gastric cancer risk: association between ZBTB20 genetic variance and gastric cancer risk in Chinese Han population[J]. Biosci Rep, 2020, 30;40(9):BSR20202102.

637. Meisami A , Jalilvand A . Association of IGF-1 gene rs2195239 polymorphism with the risk and clinical features of gastric cancer in a Chinese Han population[J]. Journal of Clinical Laboratory Analysis, 2020:e23436.

638. Li YF , Zhang Z , Yang Y , et al. Long Noncoding RNA HOX Transcript Antisense RNA Gene rs17720428 Single Nucleotide Polymorphism Is Associated with Gastric Cancer Risk and Prognosis[J]. Genetic Testing and Molecular Biomarkers, 2020, 24(1):38-46.

639. Li ZZ, Zhou LT, Zhai LH,et al. Single nucleotide polymorphism of rs28416520 in Piwil1 gene promoter region is associated with an increased risk of gastric cancer[J]. Nan Fang Yi Ke Da Xue Xue Bao, 2020 40(10):1373-1379
